# BMP4 and Gremlin 1 regulate hepatic cell senescence during clinical progression of NAFLD/NASH

**DOI:** 10.1038/s42255-022-00620-x

**Published:** 2022-08-22

**Authors:** Ritesh K. Baboota, Aidin Rawshani, Laurianne Bonnet, Xiangyu Li, Hong Yang, Adil Mardinoglu, Tamar Tchkonia, James L. Kirkland, Anne Hoffmann, Arne Dietrich, Jeremie Boucher, Matthias Blüher, Ulf Smith

**Affiliations:** 1 Lundberg Laboratory for Diabetes Research, Department of Molecular and Clinical Medicine, Sahlgrenska Academy, University of Gothenburg, Gothenburg, Sweden; 2 Wallenberg Laboratory for Cardiovascular and Metabolic Research, Institute of Medicine, University of Gothenburg, Gothenburg, Sweden; 3 Wallenberg Centre for Molecular and Translational Medicine, University of Gothenburg, Gothenburg, Sweden; 4 Science for Life Laboratory, KTH Royal Institute of Technology, Stockholm, Sweden; 5 Centre for Host-Microbiome Interactions, Faculty of Dentistry, Oral & Craniofacial Sciences, King’s College London, London, UK; 6 Department of Physiology and Biomedical Engineering, Mayo Clinic, Rochester, MN USA; 7 Helmholtz Institute for Metabolic, Obesity and Vascular Research (HI-MAG), University of Leipzig and University Hospital Leipzig, Leipzig, Germany; 8 Department of Visceral, Transplantation, Thoracic and Vascular Surgery, Section of Bariatric Surgery, University Hospital Leipzig, Leipzig, Germany; 9 Bioscience Metabolism, Research and Early Development, Cardiovascular, Renal and Metabolism (CVRM), BioPharmaceuticals R&D, AstraZeneca, Gothenburg, Sweden

**Keywords:** Biochemistry, Anatomy, Metabolism

## Abstract

The role of hepatic cell senescence in human non-alcoholic fatty liver disease (NAFLD) and non-alcoholic steatohepatitis (NASH) is not well understood. To examine this, we performed liver biopsies and extensive characterization of 58 individuals with or without NAFLD/NASH. Here, we show that hepatic cell senescence is strongly related to NAFLD/NASH severity, and machine learning analysis identified senescence markers, the BMP4 inhibitor Gremlin 1 in liver and visceral fat, and the amount of visceral adipose tissue as strong predictors. Studies in liver cell spheroids made from human stellate and hepatocyte cells show BMP4 to be anti-senescent, anti-steatotic, anti-inflammatory and anti-fibrotic, whereas Gremlin 1, which is particularly highly expressed in visceral fat in humans, is pro-senescent and antagonistic to BMP4. Both senescence and anti-senescence factors target the YAP/TAZ pathway, making this a likely regulator of senescence and its effects. We conclude that senescence is an important driver of human NAFLD/NASH and that BMP4 and Gremlin 1 are novel therapeutic targets.

## Abstract (web summary)

Baboota et al. investigate senescence as a driver of human NAFLD/NASH and show the roles of BMP4 and its antagonist Gremlin 1 as anti-senescent and pro-senescent molecules, respectively.

## Main

NAFLD has become one of the most common chronic liver diseases, with a constantly increasing prevalence worldwide due to its close association with obesity and type 2 diabetes (T2D)^1,2^. Multiple concurrent intrahepatic and extrahepatic events contribute to the development and progression of NAFLD, including insulin resistance, oxidative stress, apoptosis, cytokines and adipokines^3^. Despite its severity and high prevalence, many gaps remain in our understanding of its pathophysiology.

Cellular senescence, a state of sustained cell-cycle arrest, has attracted great attention as a potential contributor to the development of metabolic diseases and of NAFLD^4^ and NASH/cirrhosis^5^. A previous study demonstrated that senescence in hepatocytes drives hepatic steatosis in mice, whereas elimination of senescent cells prevents steatosis^6^. Increased cellular senescence in the liver has also been shown in some studies with human biopsies from patients with NAFLD/NASH^4,6^, but whether senescence is a marker or potential mediator of disease remains unclear. Moreover, key factors involved in regulating hepatocyte senescence in human NAFLD/NASH are largely unknown.

A growing number of studies have implicated the Hippo–YAP/TAZ pathway in NAFLD/NASH pathogenesis. YAP and TAZ are co-transcriptional activators that are regulated by the canonical Hippo signaling cascade^7^. Under physiological conditions, the level of YAP/TAZ phosphorylation by upstream LATS kinases maintains the balance between cytoplasmic sequestration and degradation. Activation of YAP/TAZ plays an important role in liver repair and regeneration^8,9^. However, YAP/TAZ has also emerged as important for hepatic fibrosis. YAP inactivation leads to loss of hepatocytes^10,11^ and, subsequently, liver fibrosis in vivo and in vitro^10^. Moreover, activation of the canonical Hippo pathway, resulting in YAP inactivation, has been observed in damaged hepatocytes^12^. Contrary to this, increased TAZ expression has been observed in human and murine NASH livers^13^. Also, YAP/TAZ activation has been shown to drive human stellate cell (HSC) activation and, subsequently, fibrosis^14,15^. Recently, p53-dependent cell senescence has been observed in LATS1/2-deficient mouse hepatocytes^16^, but the role of YAP/TAZ in human hepatocytes remains to be explored.

Bone morphogenetic proteins (BMPs) are members of the transforming growth factor-β (TGF-β) superfamily^17^. In recent years, a broader role for BMPs in metabolic diseases, including their role in liver health and disease, has been shown^18^, but their potential role in the development and progression of NAFLD/NASH is unclear. Increased BMP8b levels have been associated with the development of NASH^19,20^. On the other hand, BMP9 has been shown to alleviate NAFLD by improving lipid and glucose metabolism^21,22^, whereas BMP6 prevents activation of HSCs in mouse NASH models^23^. However, the potential role of BMP4 in NAFLD/NASH is unclear. A previous study demonstrated that BMP4 prevents hepatic steatosis in mice fed high-fat diet (HFD)^24^, whereas another study showed that BMP4 can activate rat HSCs^25^. HSCs play a key role in fibrosis and are subject to intricate cross-talk with neighboring cells in the liver that contribute to their maintenance of a quiescent or activated state^26^.

In the present study, we performed extensive phenotyping of 58 individuals with or without NAFLD/NASH and examined hepatic senescence and its relation to liver phenotype. We used an unbiased machine learning approach to identify key determinants of senescence in hepatocyte cells, and a three-dimensional (3D) system to characterize mechanisms and effects of senescence, as well as the role of BMP4 and its antagonist Gremlin 1 (GREM1). Both BMP4 and GREM1 are highly expressed in human liver and adipose tissue, and BMP4 exerts positive effects on insulin signaling and action, whereas GREM1 is antagonistic^18^. Here, we show that BMP4 has several novel effects in human liver cells, where it reduces cell senescence and is anti-steatotic, anti-fibrotic and anti-inflammatory. However, these positive effects are inhibited by increased GREM1, making BMP4 and GREM1 interesting novel targets in NAFLD/NASH.

## Results

We performed extensive phenotyping, including body composition, insulin sensitivity with euglycemic clamps, glucose tolerance with oral glucose load, metabolic risk factors, and cytokines, and transcriptomic analysis in liver biopsies from 58 individuals with or without a diagnosis of NAFLD and NASH. Clinical and biochemical characteristics are shown in Supplementary Table 1. We first focused on the individual characteristics of the individuals with and without NAFLD/NASH and then integrated key data to identify the best predictors of NAFLD/NASH and cellular senescence using machine learning.

### Liver senescence markers relate to severity of NAFLD/NASH

**Fig. 1:**
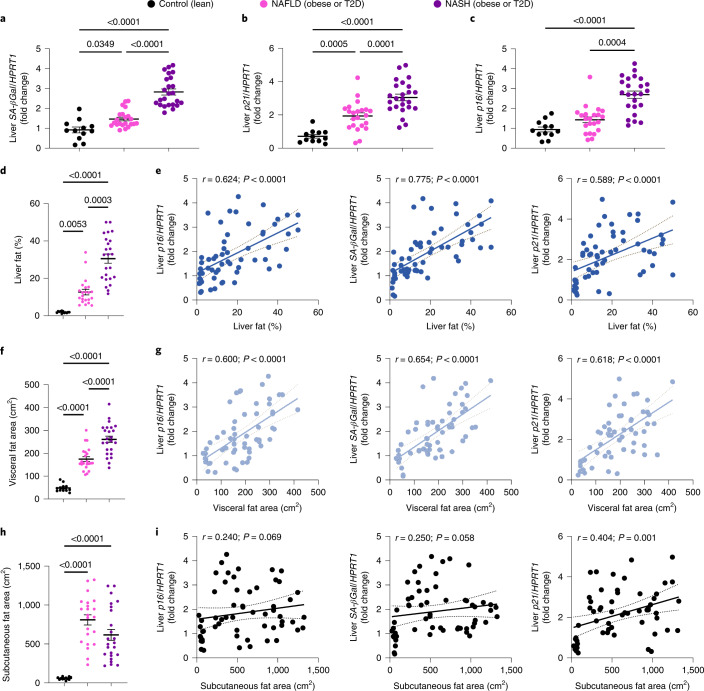
Elevated levels of hepatic senescence markers are associated with liver fat and visceral adipose tissue. **a**–**c**, RT–qPCR analysis to assess the expression levels of *SA-β-Gal* (**a**), *p21* (**b**) and *p16* (**c**) in lean individuals, patients with NAFLD and patients with NASH. **d**,**e**, Amount of liver fat (%) in lean individuals, patients with NAFLD and patients with NASH (**d**) and its correlation with mRNA levels of hepatic senescence markers (**e**). **f**,**h**, Amount of visceral (**f**) and subcutaneous (**h**) fat area in lean individuals, patients with NAFLD and patients with NASH. **g**,**i**, Correlation comparisons between mRNA levels of hepatic senescence markers and visceral fat area (**g**) or subcutaneous fat area (**i**). Data were collected in lean participants (*n* = 12), patients with NAFLD (*n* = 22) and patients with NASH (*n* = 24). Associations were determined using Pearson or Spearman correlation analysis. Values are mean ± s.e.m. Statistical significance was determined by one-way ANOVA with post hoc Tukey’s test or Kruskal–Wallis with post hoc Dunn’s test. Source data

First, we asked if key markers of cell senescence are associated with NAFLD or NASH. We examined gene expression of the established senescence markers, the cyclin D kinase inhibitors *CDKN2A* (or *p16*) and *CDKN1A* (or *p21*), as well as the lysosomal marker *GLB1* (or *SA-β-Gal*), in the liver biopsies. Compared with healthy lean individuals, all senescence markers were significantly increased in NAFLD and further upregulated in NASH individuals with a similar body mass index (BMI) (Fig. 1a–c).

**Extended Data Fig. 1:**
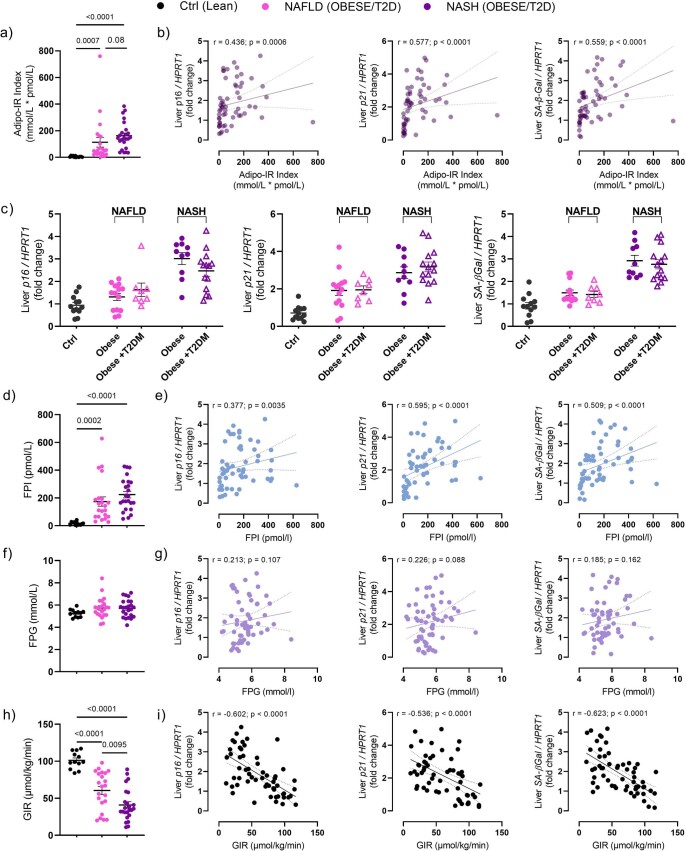
Hepatic senescence markers are associated with insulin resistance. **(a)** Adipo-IR index in lean, NAFLD, and NASH individuals. **(b)** Correlation comparisons of Adipo-IR with hepatic senescence markers. **(c)** RT-qPCR analysis of hepatic senescence markers including *p16*, *p21*, and *SA-β-Gal*, in T2D individuals compared to non-diabetic individuals within lean, NAFLD, and NASH individuals. (d, f, h) Measurement of FPI **(d)**, FPG **(f)**, and GIR **(h)** in lean, NAFLD, and NASH individuals. (e, g, i) Correlation comparisons of mRNA levels of hepatic senescence markers with FPI **(e)**, FPG **(g)**, and GIR **(i)**. Data were collected in lean subjects (n = 12), NAFLD subjects (n = 22), and NASH subjects (n = 24). Associations were determined using Spearman correlation analysis. Values are mean ± SEM. Statistical significance was determined by one-way ANOVA with *post-hoc* Tukey’s test or Kruskal-Wallis with *post-hoc* Dunn’s test. FPI, Fasting plasma insulin; FPG, fasting plasma glucose; GIR, glucose infusion rate. Source data

Amount of liver fat (%) increased with severity of liver disease (Fig. 1d) and correlated strongly with hepatic senescence markers (Fig. 1e). Visceral fat area increased with disease severity (Fig. 1f) and had strong positive correlations with all senescence markers (Fig. 1g). However, the amount of subcutaneous fat was neither different between NAFLD and NASH nor correlated with senescence markers (Fig. 1h,i). Because human adipose cells also become senescent^27,28^, we asked if senescence markers are increased in visceral or subcutaneous fat with increasing severity of disease, but no differences were observed (Supplementary Fig. 1a,b). However, detailed Kyoto Encyclopedia of Genes and Genomes (KEGG) pathway analysis of differentially expressed genes (DEGs) in visceral fat suggested that the upregulated DEGs were enriched in inflammatory pathways, including ‘primary immunodeficiency’, ‘NF-kappa B signaling’ and ‘T cell receptor signaling pathway’, whereas downregulated DEGs were significantly enriched in pathways related to metabolism, including ‘oxidative phosphorylation’, ‘thermogenesis’, ‘PPAR signaling’ and ‘insulin signaling’ pathways (Supplementary Fig. 1c). Moreover, the significant increase in adipose tissue insulin resistance index (Adipo-IR) with increasing severity of disease clearly shows adipose tissue dysfunction (Extended Data Fig. 1a). Adipo-IR also showed a strong positive correlation with hepatic senescence markers (Extended Data Fig. 1b). Thus, increased liver senescence in NAFLD/NASH is not reflected by senescence expression (per unit) of visceral fat, whereas the amount of visceral fat was closely related. Together, this indicates increased overall abundance of senescent cells due to the increased total amount of visceral fat and that the expanded visceral fat may release factors that enhance senescence in the liver cells.

We have previously found that cell senescence is increased in human adipose progenitor cells from individuals with T2D vs non-diabetic individuals^27^. Therefore, we examined if hepatic senescence is further increased in individuals with NAFLD/NASH and T2D but saw no difference compared with non-diabetic individuals (Extended Data Fig. 1c). Interestingly, fasting insulin levels were positively correlated with liver senescence (Extended Data Fig. 1d,e), but this was not seen with fasting glucose levels (Extended Data Fig. 1f,g). More importantly, degree of insulin resistance measured by the ‘gold standard’, the euglycemic glucose clamp, showed a highly consistent and significant correlation with liver senescence (Extended Data Fig. 1h,i), suggesting that hepatic senescence and associated cell secretion may promote whole-body insulin resistance.

**Fig. 2:**
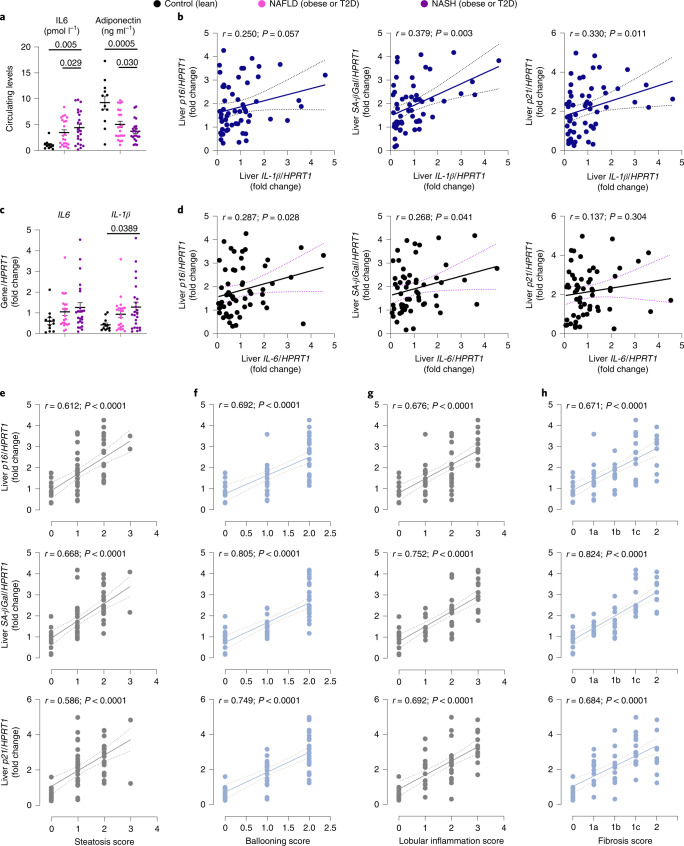
Hepatic senescence markers are associated with inflammation and hepatic fibrosis. **a**,**c**, Circulating levels of IL-6 and adiponectin (**a**), and RT–qPCR analysis of hepatic inflammatory markers *IL-1β* and *IL-6* (**c**) in lean individuals, patients with NAFLD and patients with NASH. **b**,**d**,**e**–**h**, Correlation comparisons of mRNA levels of hepatic senescence markers with *IL-1β* (**b**) and *IL-6* (**d**) mRNA levels, and with parameters of histological grading: steatosis score (**e**), ballooning score (**f**), lobular inflammation score (**g**) and fibrotic score (**h**). Data were collected in lean participants (*n* = 11–12), patients with NAFLD (*n* = 21–22) and patients with NASH (*n* = 22–24). Associations were determined using Pearson or Spearman correlation analysis. Values are mean ± s.e.m. Statistical significance was determined using Kruskal–Wallis with post hoc Dunn’s test. Source data

**Fig. 3:**
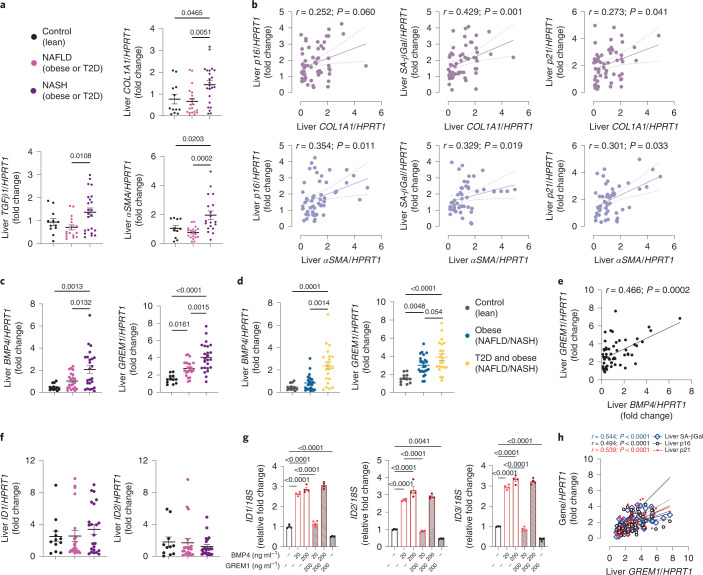
Hepatic senescence markers are associated with hepatic fibrosis. **a**, RT–qPCR analysis of hepatic fibrosis markers (*TGFβ1*, *COL1A1* and *αSMA*) in lean individuals, patients with NAFLD and patients with NASH. **b**, Correlation comparisons between hepatic senescence markers and *COL1A1* or *αSMA* mRNA levels. **c**, RT–qPCR analysis of hepatic *BMP4* and *GREM1* mRNA levels in lean individuals, patients with NAFLD and patients with NASH. **d**, RT–qPCR analysis of hepatic mRNA expression of *BMP4* and *GREM1* in lean (*n* = 12), obese (*n* = 24) and diabetic–obese (*n* = 22) individuals. **e**, Correlation between hepatic *BMP4* and *GREM1* mRNA levels. **f**, RT–qPCR analysis of hepatic *ID1* and *ID2* mRNA expression in lean individuals, patients with NAFLD and patients with NASH. **g**, RT–qPCR analysis of *ID1*, *ID2* and *ID3* mRNA levels in IHH cells including the effect of BMP4, in the presence or absence of GREM1, on expression of these genes (*n* = 4 biologically independent experiments). **h**, Correlation comparison between mRNA levels of hepatic *GREM1* and senescence markers. Data were collected in lean participants (*n* = 11–12), patients with NAFLD (*n* = 17–22) and patients with NASH (*n* = 19–24). Associations were determined using Spearman correlation analysis. Values are mean ± s.e.m. Statistical significance was determined by one-way ANOVA with post hoc Tukey’s test or Kruskal–Wallis with post hoc Dunn’s test. Source data

Consistent with this, hepatic messenger RNA and circulating levels of different cytokines were increased, whereas adiponectin (a positive marker of insulin sensitivity) was decreased in NAFLD/NASH (Fig. 2a,c). Of note, hepatic mRNA levels of the SASP cytokines, *IL-1β* and *IL-6*, were also related to the degree of senescence (Fig. 2b,d). We analyzed the relationship between senescence markers and severity of NAFLD/NASH determined by the pathological scoring of hepatic steatosis, ballooning, lobular inflammation and fibrosis. All three senescence markers showed strong positive associations with the different scores (Fig. 2e–h). Moreover, genes involved in hepatic fibrosis (that is, *TGFβ1*, *COL1A1* and *αSMA*) were markedly increased in individuals with NASH compared with lean individuals or those with NAFLD (Fig. 3a) and were also positively correlated with senescence markers (Fig. 3b).

Collectively, these results provide evidence that liver cell senescence is increased in NAFLD, related to liver lipid levels, further increased in NASH, and related to pathological severity. Furthermore, both inflammatory and fibrotic markers were also related to the extent of senescence. Additionally, we used a public database of 206 patients that validated our findings of increased hepatic senescence in NAFLD (Supplementary Data 2a).

### Hepatic BMP4 and GREM1 expression in patients with NAFLD/NASH

Compared with lean individuals, both hepatic *BMP4* and *GREM1* mRNA levels are higher in NAFLD and further increased in NASH (Fig. 3c), and further increased in individuals with T2D (Fig. 3d). Unexpectedly, there was a strong positive correlation between hepatic *BMP4* and *GREM1* (Fig. 3e). We also verified our findings of increased hepatic BMP4 and GREM1 in NAFLD/NASH in a large publicly available cohort (Supplementary Data 2a).

The close correlation between hepatic *GREM1* and *BMP4* suggests that GREM1 might inhibit effects of BMP4. To investigate this, we measured the expression of *ID1* and *ID2*, early downstream markers of the BMP signaling cascade^29^. Compared with lean individuals, we observed no change in expression of *ID1* and *ID2* in NAFLD or NASH, in spite of their higher *BMP4* levels (Fig. 3f). We confirmed these effects in hepatocytes in vitro. The addition of BMP4 to immortalized human hepatocyte (IHH) cells significantly increased *ID1*, *ID2* and *ID3* mRNA levels, whereas this increase was prevented by exogenous GREM1 (Fig. 3g).

Next, we examined if the increased *GREM1* in NAFLD and NASH is associated with the increased liver senescence. Indeed, there was a strong positive correlation between hepatic *GREM1* and the senescence markers *p21*, *p16* and *SA-β-Gal* in our clinical cohorts, suggesting a role for GREM1 and BMP4 in regulating senescence (Fig. 3h), which we later also validated. We further observed that hepatic *GREM1* levels were positively correlated with several markers of senescence in NAFLD/NASH, including visceral fat area, insulin levels and insulin resistance measured using homeostatic model assessment for insulin resistance (HOMA-IR), Adipo-IR and inflammatory (serum and liver interleukin-6 (IL-6) levels) and hepatic fibrotic (*αSMA* and *COL1A1*) markers, whereas they were negatively correlated with serum adiponectin levels (Supplementary Table 2).

### Single-cell RNA-seq analysis in major hepatic cell types

To explore the expression of hepatic senescence markers (*p21*, *p16* and *SA-β-Gal*), *BMP4* and *GREM1* within specific cell populations, we examined publicly available single-cell RNA sequencing (RNA-seq) data of healthy and cirrhotic human livers (which includes one patient with NAFLD)^30^. The senescence markers were expressed in multiple cell types, but the percentage of cells expressing these markers varies among the cell types (Supplementary Data 2b). For instance, the percentage of *p21-*positive cells was high among different cell types in comparison with *p16-*positive and *SA-β-Gal-*positive cells. *p21-*positive cells were mostly observed in plasma, mesenchymal, and endothelial cells, as well as plasmacytoid dendritic cells, and to lesser extent in mononuclear phagocytes, hepatocytes or cholangiocytes, and cycling cells (Supplementary Data 2b). However, in cirrhotic liver, the percentage of *p21-*positive cells tends to increase only in hepatocytes or cholangiocytes, plasmacytoid dendritic cells and mononuclear phagocytes (Supplementary Data 2c). Similarly, the percentage of *SA-β-Gal-*positive cells was also highly observed in cycling cells, plasma cells and mononuclear phagocytes, whereas under a cirrhotic state, the percentage of *SA-β-Gal-*positive cells increased only in hepatocytes or cholangiocytes and mononuclear phagocytes. More importantly, *p16-*positive cells, which were found in cycling and plasma cells, but not in hepatocytes or cholangiocytes in healthy liver, tend to increase in hepatocytes or cholangiocytes in cirrhotic liver (Supplementary Data 2c). Additionally, BMP4 was observed mainly in hepatocytes or cholangiocytes and endothelial cells. The percentage of BMP4-positive cells was significantly increased in endothelial and mesenchymal cells (*P*_adj_ < 0.05) in cirrhosis and to some extent in hepatocytes or cholangiocytes. On the other hand, a low percentage of GREM1-positive cells was observed among all cell types and was not increased much in cirrhotic liver (Supplementary Data 2b,c).

It should be emphasized that there are clear limitations in the interpretation of single-cell data. Patients with cirrhotic livers do not truly reflect early NAFLD etiology. This study used five cirrhotic livers, which included two livers from patients with NAFLD, two livers from patients with alcohol-related liver disease and one liver from a patient with primary biliary cholangitis. Moreover, out of the two NAFLD samples, only one sample had the sequencing data from non-parenchymal cells, leaving us with only one NAFLD sample. Taken together, these findings indicate that different cell types might be involved in hepatic senescence. However, future prospective studies with larger numbers of samples are required to better understand the roles of the different cells in NAFLD/NASH.

### Effect of doxorubicin-induced senescence in IHH cells

**Fig. 4:**
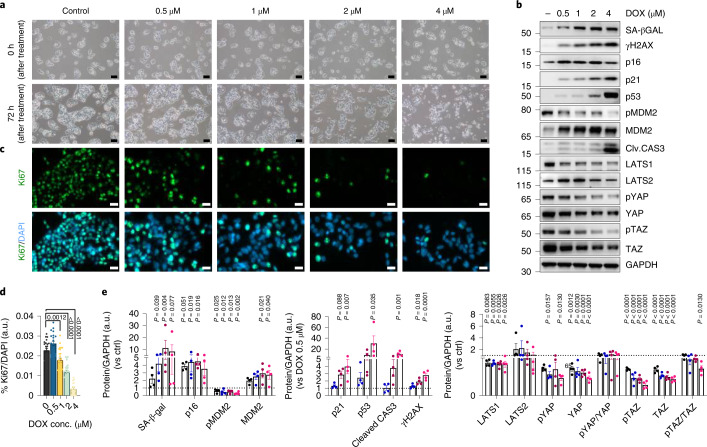
DOX-induced senescence in IHH cells. **a**, Representative images (bright-field) of IHH cells treated with different concentrations of DOX for 72 h (*n* = 3 biologically independent experiments). Scale bars represent 100 µm. **b**, Representative immunoblots of respective proteins in control and DOX-treated cells after 72 h (*n* = 4 biologically independent experiments). **c**, Representative immunofluorescence images of control and DOX-treated cells stained for Ki67 (green) and nuclei (DAPI, blue) (*n* = 3 biologically independent experiments). Scale bars represent 20 µm. **d**, Bar graphs showing Ki67 positivity in control and DOX-treated hepatocytes (*n* = 3, 7–10 randomly chosen fields from each experiment). **e**, Bar graphs showing the relative protein levels of senescence markers, as well as markers of the YAP/TAZ pathway, in control and DOX-treated cells, normalized to GAPDH (*n* = 4 biologically independent experiments). Values are mean ± s.e.m. Statistics were calculated using one-way ANOVA followed by Dunnett’s post hoc test or Kruskal–Wallis with post hoc Dunn’s test. a.u., arbitrary unit. Source data

**Extended Data Fig. 2:**
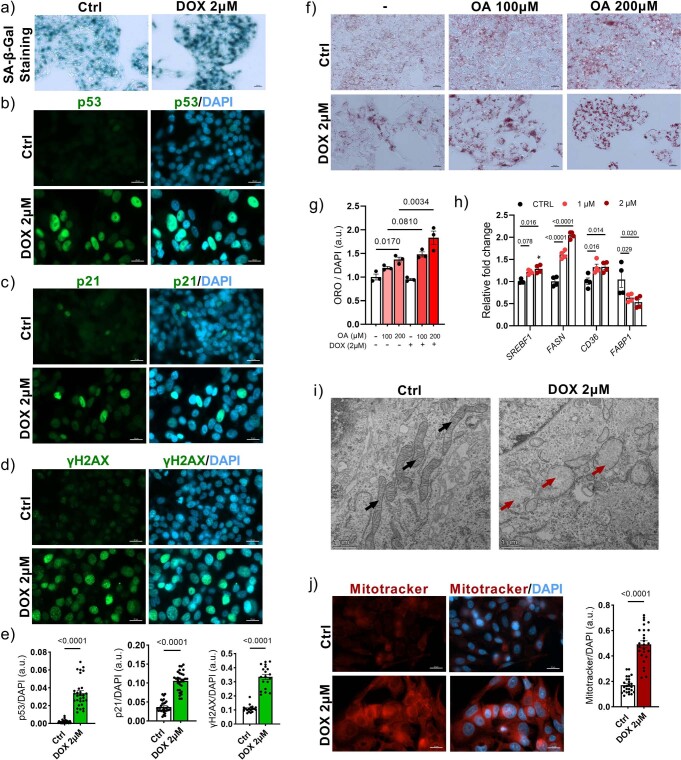
Doxorubicin-induced senescence in IHH cells. **(a)** Brightfield microscopy images of control and DOX-treated cells assayed for SA-β-Gal (blue). Scale bar represents 100 µm. (b-d) Representative immunofluorescence images of control and DOX-treated cells stained for p53 (green) **(b)**, p21 (green) **(c)**, γH2AX (green) **(d)**, and nuclei (DAPI, blue). Scale bar represents 20 µm. **(e)** Bar graph displays fluorescence intensities quantified using ImageJ and normalized to number of nuclei (n = 3, 6-10 randomly chosen fields from each experiment). **(f)** After 48 h, DOX-treated IHH cells were stimulated with OA for 24 h. Cells were stained for lipid droplet accumulation (ORO, red) and nuclei (DAPI). Scale bar represents 100 µm. **(g)** Intracellular lipid accumulation was quantified by spectrophotometry and normalized to number of nuclei (n = 3). **(h)** RT-qPCR analysis of genes involved in lipid metabolism in DOX-treated cells (n = 4). **(i)** Representative transmission electron micrographs of control and DOX-treated hepatocytes. Scale bar represents 1 μm. (n = 3 biologically independent experiments). **(j)** Representative fluorescence images of control and DOX-treated cells stained with Mitotracker Red dye (mitochondria, red), which detects mitochondrial polarization status. Scale bar represents 20 μm. Bar graph displays fluorescence intensities of Mitotracker Red, normalized to number of nuclei (n = 3, 6-10 randomly chosen fields from each experiment). Values are mean ± SEM. Statistics: 2-tailed, Unpaired t-test for **(e)** (p21 & γH2AX), Mann-Whitney test for (e, j) (p53 & Mitotracker), and one-way ANOVA followed by Bonferroni’s/Dunnett’s *post-hoc* test or Kruskal-Wallis with *post-hoc* Dunn’s test (g, h). Black arrows indicate normal mitochondria and red arrows indicate mitochondria with disrupted cristae. a.u., arbitrary unit. Source data

To examine mechanisms and consequences of hepatocyte senescence, we incubated human IHH cells with the chemotherapeutic agent doxorubicin (DOX), a well-established inducer of premature senescence^31,32^. First, we established appropriate incubation conditions by treating the cells with different concentrations of DOX for 2 h, followed by incubation with fresh medium without DOX for 72 h to ensure a lasting effect. A concentration of 4 µM was too high, with increased cleaved caspase-3 expression (Fig. 4b,e) and increased cell death (Fig. 4a). However, the cells became senescent as documented by enlarged and flattened cell morphology (Fig. 4a); a decreased number of Ki67-positive cells; reduced proliferation (Fig. 4c,d); increased protein levels of SA-β-Gal, γH2AX, p16, p21 and p53; reduced phosphorylation of MDM2 and increased MDM2 (Fig. 4b,e). Similar senescence effects were seen with lower DOX concentrations (that is, 1 μM and 2 μM), except for increased cleaved caspase-3 levels (Fig. 4b,e); therefore, 2 μM was chosen for further experiments. This resulted in increased SA-β-Gal activity in these cells (Extended Data Fig. 2a), and increases in p53 and p21 were confirmed using immunostaining (Extended Data Fig. 2b,c,e). Senescent cells had puncta of staining signals for γH2AX (Extended Data Fig. 2d,e), typical for senescence-associated DNA damage.

We evaluated effects of DOX-induced senescence on lipid accumulation by incubating senescent cells with oleic acid. Oil Red O (ORO) staining clearly demonstrated increased lipid accumulation compared with control (Extended Data Fig. 2f,g). An increase in *CD36*, *SREBF1* and *FASN* mRNA levels, but a decrease in *FABP1*, was also observed (Extended Data Fig. 2h). Furthermore, transmission electron microscopy (TEM) imaging clearly revealed that, unlike the control cells with well-packed stacks of mitochondrial cristae, the senescent cells exhibited incomplete and disrupted cristae (Extended Data Fig. 2i). Mitochondrial membrane potential was measured using MitoTracker Red CMXRos, which revealed enhanced intensity in senescent cells consistent with hyperpolarized mitochondria (Extended Data Fig. 2j). Thus, DOX treatment markedly induces senescence in the IHH cells associated with mitochondrial dysfunction and increased lipid accumulation.

### Effect of BMP4 and GREM1 on cell senescence and YAP/TAZ

**Extended Data Fig. 3:**
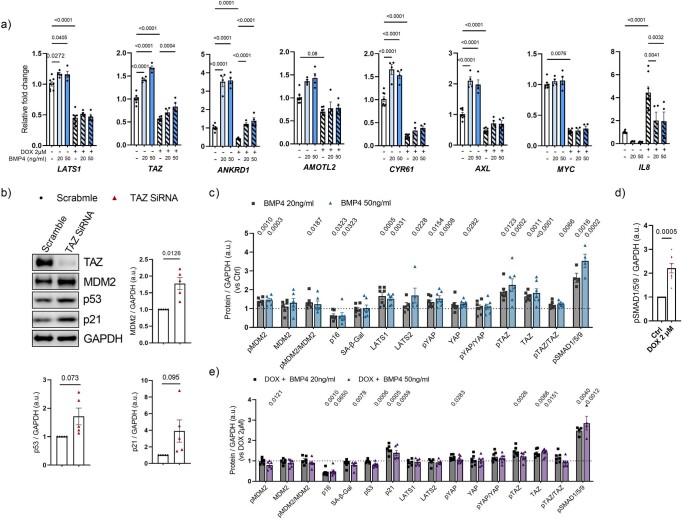
Effects of BMP4 in DOX-treated IHH cells. **(a)** RT-qPCR analysis of genes involved in the YAP/TAZ pathway and inflammation in control and DOX-treated cells, stimulated with or without BMP4 (n = 4). **(b)** Representative immunoblots of proteins (p53, p21, and MDM2), and their quantification by densitometry (bar graphs) in siRNA-transfected IHH cells. (c-e) Bar graphs showing expression of respective proteins in control cells (with or without BMP4, n = 6) **(c)** and DOX-treated cells (with or without BMP4, n = 4) (**d, e**). Values are mean ± SEM. Statistics: One-way ANOVA followed by Bonferroni’s/Dunnett’s *post-hoc* test or Kruskal-Wallis with *post-hoc* Dunn’s test for (a, c, e), 2-tailed paired t-test for (b, d). a.u., arbitrary unit. Source data

Immunoblot analysis revealed that protein levels of YAP1 (YAP) and WWTR1 (TAZ) were markedly reduced and there was a decrease in Hippo-induced phosphorylated YAP (pYAP) and TAZ (pTAZ) with increasing concentrations of DOX (Fig. 4b,e). Evaluation of the tumor suppressors, LATS1/2, the direct upstream regulators of Hippo/YAP and TAZ, showed that LATS1 was significantly reduced, whereas LATS2 was not affected (Fig. 4b,e). *LATS1* and *TAZ* mRNA levels were also significantly downregulated (Extended Data Fig. 3a). TAZ is an inhibitor of p53 and senescence^33^, and we also observed an enhanced expression of senescence markers, including p53, MDM2 and p21, following TAZ silencing in IHH cells (Extended Data Fig. 3b). Thus, the reduction in TAZ is one important factor promoting senescence by DOX in human hepatocytes, and the expression of the well-characterized YAP/TAZ target genes (that is, *ANKRD1*, *AXL*, *AMOTL2*, *CYR61* and *MYC*) was also significantly reduced (Extended Data Fig. 3a).

**Fig. 5:**
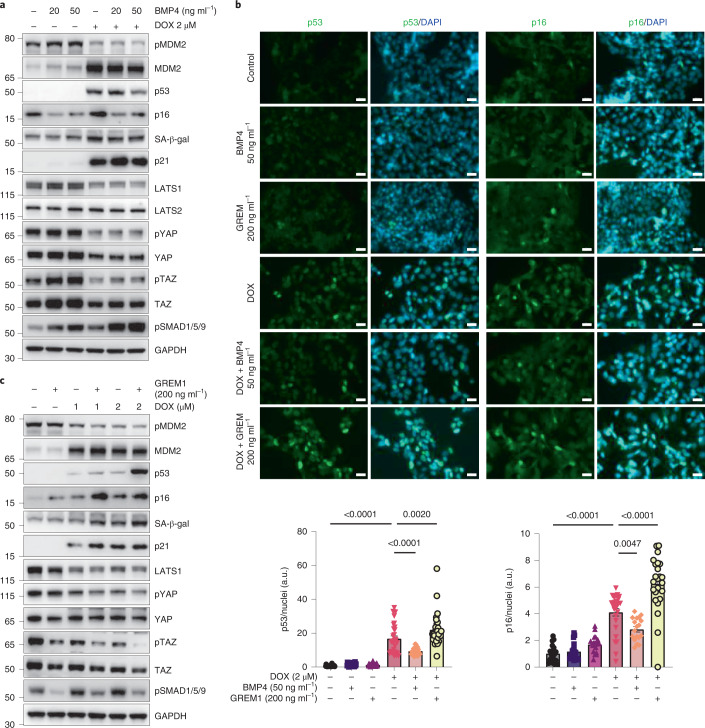
BMP4 prevents the increase in DOX-induced senescence markers, whereas GREM1 enhances the effects of DOX. **a**,**c**, Representative immunoblots of respective proteins in control and DOX-treated cells stimulated with or without BMP4 (20 ng ml^−1^ and 50 ng ml^−1^) (**a**) or GREM1 (200 ng ml^−1^) (**c**) (*n* = 6 or 7 biologically independent experiments, except for LATS2 (*n* = 5) and pSMA1/5/9 (*n* = 4)). **b**, Representative immunofluorescent images of control and DOX-treated cells, stimulated with or without BMP4 or GREM1, stained for p53 (green), p16 (green) and nuclei (DAPI, blue). Scale bars represent 20 µm. Bar graph displays fluorescence intensities quantified using ImageJ and normalized to the number of nuclei (*n* = 3, 6–10 randomly chosen fields from each experiment). Values are mean ± s.e.m. Statistics were calculated using one-way ANOVA followed by Bonferroni’s post hoc test. Source data

**Extended Data Fig. 4:**
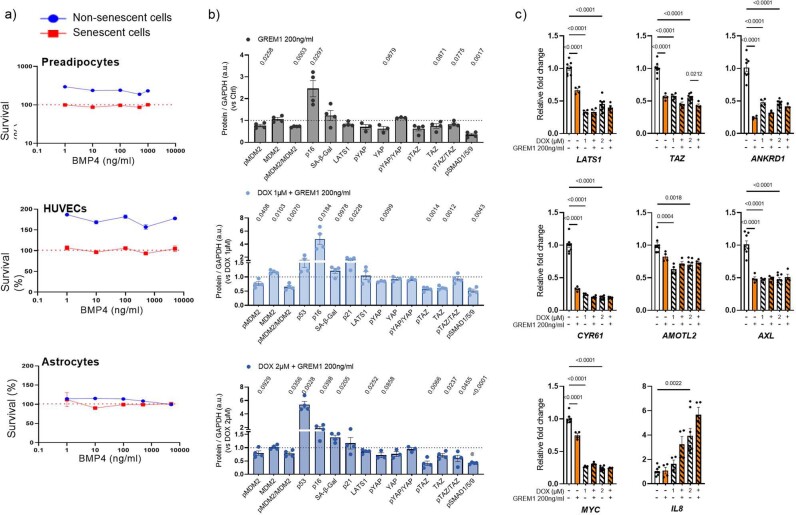
Effects of GREM1 in DOX-treated IHH cells. (a) Quantification of cell viability of non-senescent and induced senescent cells (preadipocytes, HUVECs, and astrocytes) incubated with increasing concentrations of BMP4 (n = 5). (b) Bar graphs showing expression of respective proteins in control cells (with or without GREM1, n = 4), DOX (1 µM)-treated cells (with or without GREM1, n = 4), and DOX (2 µM)-treated cells (with or without GREM1, n = 4) (h). (c) RT-qPCR analysis of respective genes involved in the YAP/TAZ pathway and inflammation in control and DOX-treated cells, stimulated with or without GREM1 (n = 4). Values are mean ± SEM. Statistics were calculated using Mann-Whitney test for (b) and one way ANOVA followed by Bonferroni’s *post-hoc* test or Kruskal-Wallis with *post-hoc* Dunn’s test for (c). a.u., arbitrary unit. Source data

Next, we investigated effects of BMP4 and GREM1 in senescent hepatocytes. BMP4 significantly enhanced protein levels of the tumor suppressor LATS1, as well as pTAZ and total TAZ, but only slightly increased pYAP and YAP (Fig. 5a and Extended Data Fig. 3c). Intriguingly, the CDK inhibitor p16 was markedly reduced by BMP4, and importantly, BMP4 prevented the increase in SA-β-Gal, p16 and p53 levels in DOX-treated hepatocytes (Fig. 5a and Extended Data Fig. 3c,e). These preventive effects by BMP4 on p16 and p53 were further confirmed by immunostaining (Fig. 5b). BMP4 also significantly prevented the decrease in both pTAZ and TAZ in DOX-treated cells but had only minor effects on pYAP (Fig. 5a and Extended Data Fig. 3e). Gene expression analysis further corroborated these data, that is, BMP4 enhanced mRNA levels of *LATS1*, *TAZ* and its target genes and also prevented the DOX-induced reduction in *TAZ* and *ANKRD1* (Extended Data Fig. 3a). Interestingly, BMP4 significantly reduced DOX-induced *IL-8* expression, suggesting that BMP4 is anti-inflammatory (Extended Data Fig. 3a). Together, these data showed that BMP4 reduces or prevents induction of senescence in IHH cells. This effect of BMP4 is not due to a senolytic effect (that is, directly killing senescent cells), as we tested this effect in three different human cell types, including preadipocytes, human umbilical vein endothelial cells (HUVECs) and astrocytes (Extended Data Fig. 4a).

In contrast to the effects of BMP4, GREM1 was pro-senescent. GREM1 enhanced the effects of DOX on the YAP/TAZ pathway, significantly increased p16 and reduced LATS1, pTAZ and TAZ protein (Fig. 5c and Extended Data Fig. 4b); mRNA levels of *LATS1*, *TAZ* and its target genes were also reduced (Extended Data Fig. 4c). GREM1 markedly enhanced the pro-senescent effects of DOX and increased p16, p53 and SA-β-Gal protein (Fig. 4c). The GREM1-mediated increase in p16 and p53 in DOX-induced senescent cells was confirmed by immunostaining (Fig. 5b).

As expected, the increase in pSMAD1/5/9 levels by BMP4 (Fig. 5a and Extended Data Fig. 3c) was clearly inhibited by GREM1 (Fig. 5b and Extended Data Fig. 4b), whereas DOX itself significantly increased pSMAD1/5/9 (Extended Data Fig. 3d), which could be a result of DNA damage resulting in activated TGF-β signaling^34^.

Collectively, these data provide evidence that BMP4 exerts anti-senescent effects in human IHH cells with reduction of p16 and p53 and activation of the Hippo pathway with increased LATS1, pTAZ and TAZ, whereas YAP is not a clear target. TAZ is a suppressor of p53^33^, which may be one important effect of BMP4. On the other hand, GREM1 is pro-senescent and increases p16, reduces pTAZ and TAZ and enhances the senescence-inducing effects of DOX.

### BMP4 reduces fibrotic and inflammatory markers in spheroids

**Fig. 6:**
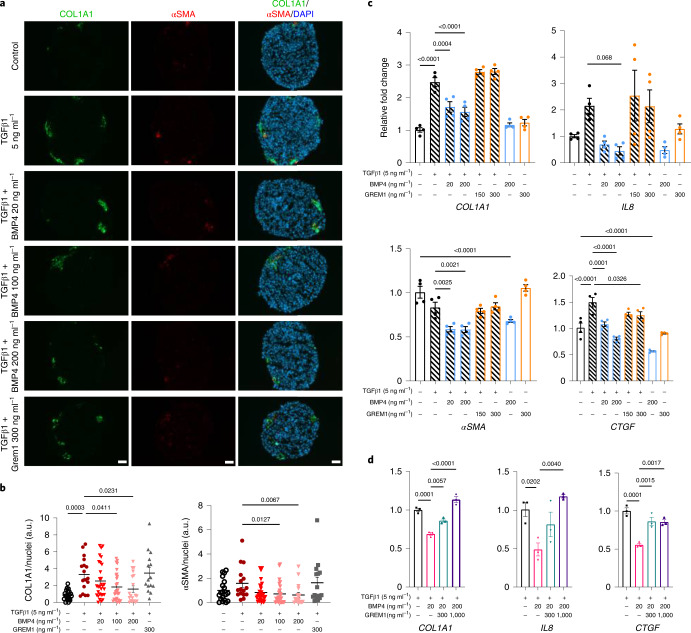
BMP4 reduced liver fibrogenic and inflammatory markers in TGF-β1-induced 3D spheroids. **a**, Representative immunofluorescence images of 3D spheroids (IHH/LX-2, 24:1), treated with TGF-β1 in presence or absence of BMP4 or GREM1 for 48 h, stained for COL1A1 (green), αSMA (red) and nuclei (DAPI, blue). Scale bars represent 50 µm. **b**, Bar graph displays fluorescence intensities of COL1A1 and αSMA quantified using ImageJ and normalized to the number of nuclei (*n* = 15–30 spheroids from three different experiments). **c**, RT–qPCR analysis of *COL1A1*, *αSMA*, *IL-8* and *CTGF* in 3D spheroids, treated with TGF-β1 in presence or absence of BMP4 (*n* = 4 biologically independent experiments). **d**, RT–qPCR analysis of *IL-8, CTGF* and *COL1A1* in TGF-β1-treated 3D spheroids, stimulated with BMP4 in presence or absence of GREM1 (*n* = 3 biologically independent experiments). Values are mean ± s.e.m. Statistical significance was determined using one-way ANOVA followed by Bonferroni post hoc test or Kruskal–Wallis with post hoc Dunn’s test. Source data

**Extended Data Fig. 5:**
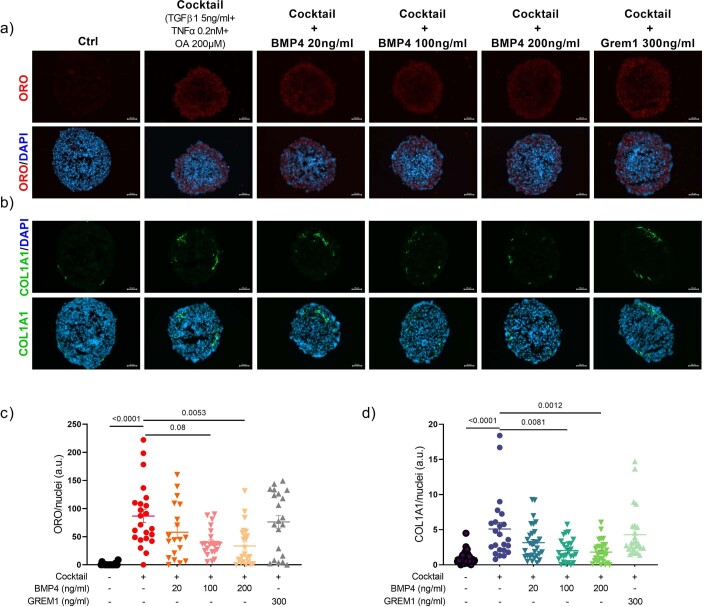
Effects of BMP4 in cocktail-induced 3D spheroids. (**a, b**) Representative immunofluorescence images of 3D spheroids (IHH/LX-2, 24:1) treated with cocktail (oleic acid (200 µM) + TGFβ1 (5 ng/ml) + TNFα (0.2 nM)) in presence or absence of BMP4/GREM1 for 48 h, stained for ORO (red) **(a)**, COL1A1 (green) **(b)**, and nuclei (DAPI, blue). Scale bar represents 50 µm. (c, d) Bar graph displays fluorescence intensities of ORO **(c)** and COL1A1 **(d)** quantified using ImageJ and normalized to number of nuclei (n = 20-30 spheroids from 3 different experiments). Values are means ± SEM. Statistical significance was determined using Kruskal-Wallis with *post-hoc* Dunn’s test. a.u., arbitrary unit. Source data

To mimic the in vivo microenvironment, human hepatocytes were incubated together with HSCs (LX-2 cells) as a multi-lineage spheroid representing a physiological in vitro model system. Exposure of spheroids to TGF-β1 for 48 h significantly enhanced both mRNA and protein levels of COL1A1 (Fig. 6a–c), together with some increase in αSMA protein (Fig. 6a,b). In a concentration-dependent manner, BMP4 significantly prevented the increase in COL1A1 and αSMA protein (Fig. 6a,b), and mRNA levels were also reduced (Fig. 6c). Additionally, the TGF-β1-mediated increase in pro-fibrogenic and pro-inflammatory markers, *CCN2* (*CTGF*)^35^ and *CXCL8* (*IL-8*)^36^, was significantly prevented by BMP4 (Fig. 6c). GREM1 did not exert any direct effect on these genes, but it significantly prevented the BMP4-mediated reduction in *COL1A1*, *IL-8* and *CTGF* (Fig. 6d). Spheroids were also exposed to a cocktail of lipogenic (oleic acid) and inflammatory triggers (TGF-β1 and tumor-necrosis factor-α, TNF-α) to mimic different aspects of the disease. Intriguingly, BMP4 significantly prevented the increase in lipid accumulation and COL1A1 protein in a dose-dependent manner, whereas GREM1 did not exert any direct effect (Extended Data Fig. 5a–d).

**Extended Data Fig. 6:**
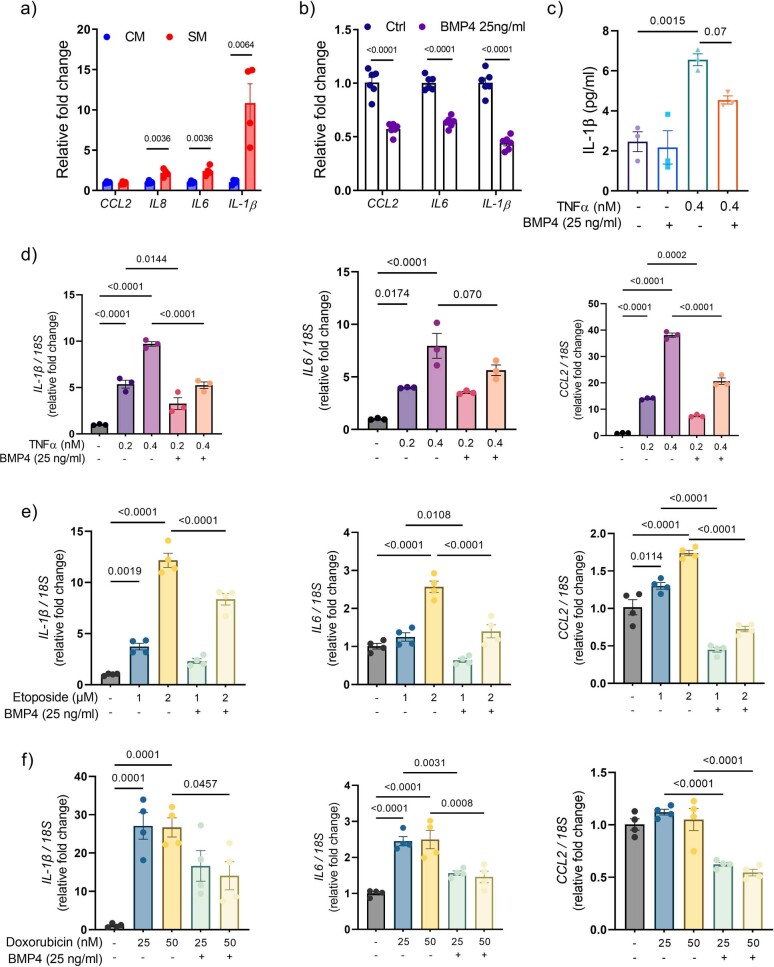
BMP4 inhibits the expression of pro-inflammatory markers. **(a)** RT-qPCR analysis of *CCL2*, *IL8*, *IL6*, and *IL-1β* in LX2 cells treated with conditioned medium (50%; *v*/*v*) from control or DOX-treated IHH cells. **(b)** Bar graph displays BMP4 *per se* effects on mRNA levels of inflammatory genes (*CCL2*, *IL-6*, and *IL-1β)* (n = 3). **(c)** Quantification of IL-1β secretion in supernatant of LX2 cells treated with TNFα in presence or absence of BMP4 (n = 3). (**d-f**) RT-qPCR analysis of inflammatory genes (*CCL2*, *IL-6*, and *IL-1β)* in LX2 cells treated with TNFα **(d)**, Etoposide **(e)**, or DOX **(f)** in presence or absence of BMP4 for 48 h. Values are as mean ± SEM. Statistics: Statistical significance was determined using 2-tailed unpaired t-test for (a, f) and one-way ANOVA followed by Bonferroni’s *post-hoc* test for (b-e, g, h). a.u., arbitrary unit. Source data

As senescent cells trigger senescence in neighboring cells via enhanced secretion of senescence-associated secretory phenotype (SASP) factors, it is necessary to suppress SASP factors in order to attenuate the paracrine spread of senescence. Therefore, we first confirmed the effects of hepatocyte SASP factors on HSCs by treating them with conditioned media from DOX-induced senescent hepatocytes. Indeed, senescent media clearly enhanced the expression of pro-inflammatory genes (*IL-8*, *IL-6* and *IL-1β*) in HSCs (Extended Data Fig. 6a). Also, senescent HSCs have been shown to contribute to hepatocellular carcinoma development through their secretion of SASP factors^37^. Therefore, we used different insults (that is, inflammatory cytokine and senescence-inducing drugs) to induce inflammation in HSCs and studied the effects of BMP4 and GREM1. TNF-α, etoposide and DOX markedly enhanced expression of the pro-inflammatory cytokines *CCL2*, *IL-6* and *IL-1β* (Extended Data Fig. 6d–f) and also increased TNF-α-mediated IL-1β secretion (Extended Data Fig. 6c). However, BMP4 significantly reduced mRNA levels of these cytokines (Extended Data Fig. 6b) and prevented the increase in mRNA and IL-1β secretion in HSCs treated with different insults (Extended Data Fig. 6c–f). Taken together, these results show that BMP4 is anti-steatotic, anti-inflammatory and anti-fibrotic, whereas GREM1 antagonizes the effect of BMP4.

### RNA-seq: signaling pathways targeted by GREM1

**Extended Data Fig. 7:**
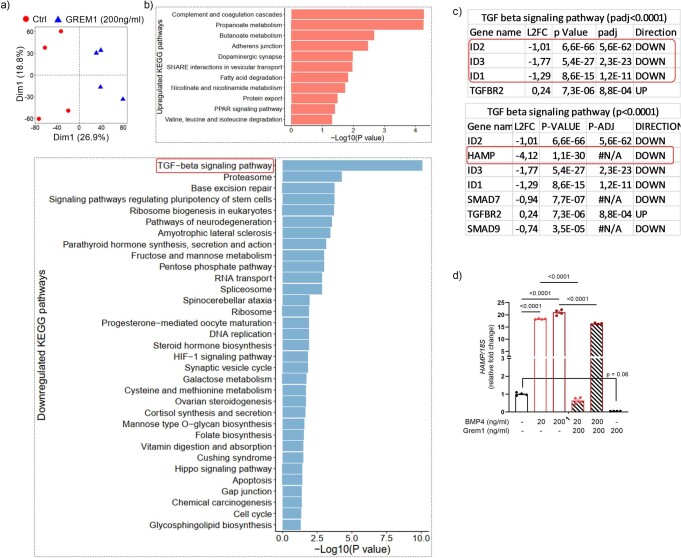
RNA-seq analysis of GREM1-treated IHH cells. **(a)** Principal component analysis representing RNA-seq expression data from all biological replicates. **(b)** RNA-seq was performed in IHH cells treated with GREM1 for 24 h (n = 4) and subsequently, KEGG pathway analysis was performed on DEGs with p < 0.05 and is represented as upregulated and downregulated pathways. **(c)** List of genes involved in TGF beta signaling pathway. Significant genes with padj<0.0001 and p < 0.0001 are included in the list. **(d)** RT-qPCR validation of RNA-seq data for *HAMP* gene, including the effect of BMP4, in presence or absence of GREM1, on the mRNA level of these target genes (n = 4). Values are mean ± SEM. Statistical significance was determined using one-way ANOVA followed by Bonferroni *post-hoc* test. Source data

As GREM1 has interesting senescence-promoting effects, we performed RNA-seq of GREM1-treated IHH cells to identify novel genes and their associated pathways. The principal component analysis (PCA) plot showed good separation between the control and GREM-treated cells, suggesting distinct gene expression patterns (Extended Data Fig. 7a). A total of 201 DEGs were identified (*P*_adj_ ≤ 0.05; fold change ≥ 1.2) (Supplementary Table 3). KEGG pathway analysis of DEGs (*P* ≤ 0.05) revealed that upregulated genes were enriched in 11 pathways, whereas downregulated genes were enriched in 33 pathways (Extended Data Fig. 7b). However, the most significantly enriched pathway, ‘TGF beta signaling’, is enriched with downregulated genes, including mainly *ID1*, *ID2* and *ID3* (Extended Data Fig. 7c), supporting its inhibition of BMP signaling. Interestingly, analysis of DEGs (using *P* ≤ 0.0001 as the criterion) clustered in the ‘TGF beta signaling’ led to the identification of an important target gene, *HAMP* (hepcidin) (Extended Data Fig. 7c). *HAMP*, a peptide hormone produced by hepatocytes, has been shown to prevent HSC activation with subsequent attenuation of liver fibrosis^38^. Downregulation of genes including *ID1–ID3* (Fig. 3g) and *HAMP* (Extended Data Fig. 7d) was validated by quantitative PCR (qPCR). Moreover, we showed that the BMP4-induced increase in expression of *ID1–ID3* (Fig. 3g) and *HAMP* (Extended Data Fig. 7d) was clearly inhibited by GREM1. Other known genes upregulated by GREM1 include genes associated with hyperglycemia (*DUSP6*^39^), NAFLD/NASH (*FABP1*^40,41^, *LYZ*^42^ and *TP53INP1*^43^), liver fibrosis (*CTHRC1*^44^) and hepatocellular carcinoma (*LPAR6*^45^ and *CCL15*^46^) (Supplementary Table 3).

### Machine learning identifies the best predictors of NAFLD/NASH

We used machine learning to identify feature importance and interactions for NAFLD/NASH and GREM1/BMP4. The complete variable list of baseline characteristics for predictors used in the statistical models is presented in Supplementary Table 4.

#### NAFLD/NASH

**Fig. 7:**
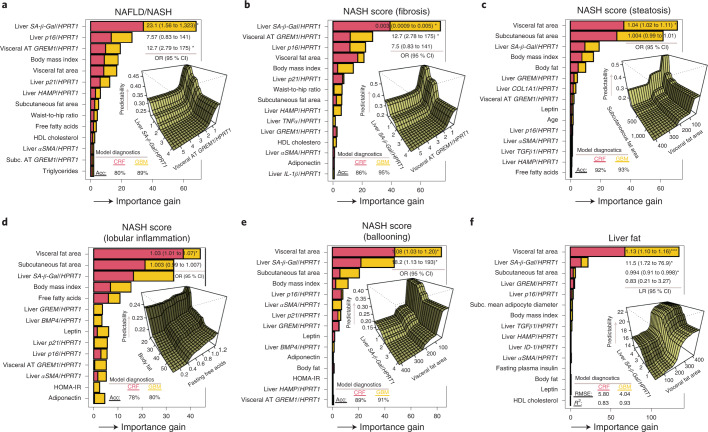
Strength of association of selected predictors for features of NAFLD/NASH and liver fat in patients with NAFLD/NASH. **a**–**f**, Relative feature importance for NAFLD/NASH features (including steatosis, ballooning, inflammation and fibrosis score) and liver fat using predictive machine learning models such as conditional random forest, gradient boosting models and partial dependence plots. Predictors that display a pronounced increase in relative importance are considered strong predictors for the outcome. Model diagnostics (that is, *R*^2^ and r.m.s.e.) for predictive machine learning models are presented in each panel. Partial dependence plots were used to investigate interaction effects between important features. To assess significance level and estimate risk association for important features, according to machine learning models, we subsequently constructed either a linear or logistic regression model and included the most important features. AT, adipose tissue.

Prediction models for NAFLD/NASH, shown in Fig. 7a, revealed that the strongest features were liver senescence-associated *SA-β-Gal*, *p16* and visceral adipose tissue *GREM1*. Logistic regression indicated that *SA-β-Gal* (odds ratio, 23.1; 95% confidence interval (CI), 1.56 to 1,323.0) and visceral *GREM1* (odds ratio, 12.7; 95% CI, 2.79 to 175.0) were significant predictors for NAFLD/NASH (Fig. 7a). Partial dependence analysis (Fig. 7a, center right) indicated that higher levels of *SA-β-Gal* and visceral *GREM1* increase predictability for NAFLD/NASH.

#### Degree of fibrosis in NASH

*SA-β-Gal*, visceral *GREM1*, liver *p16* and visceral fat area were the strongest predictors (Fig. 7b), and logistic regression revealed that *SA-β-Gal* and visceral *GREM1* were significantly associated with extent of fibrosis. Partial dependence analyses (Fig. 7b) revealed similar interaction patterns for those observed in the NAFLD/NASH model.

#### Degree of steatosis, lobular inflammation and ballooning in NASH

Pathologist-determined degree of steatosis (Fig. 7c), lobular inflammation (Fig. 7d) and ballooning (Fig. 7e) were predicted by visceral fat area, subcutaneous fat area, *SA-β-Gal* and general markers of adiposity. For the degree of steatosis, logistic regression demonstrated that visceral fat area was statistically significant. Regression (Fig. 7d) indicated that visceral fat area was the only significant predictor for degree of lobular inflammation, whereas the model for ballooning (Fig. 7e) showed that both visceral fat area and *SA-β-Gal* were statistically significant.

#### Liver fat

Liver fat was best predicted by visceral fat area and *SA-β-Gal* (Fig. 7f). This was verified in the partial dependence analysis, in which *SA-β-Gal* and visceral fat were strong interacting predictors (Fig. 7f). Linear regression indicated that visceral fat, *SA-β-Gal* and subcutaneous fat area were statistically significant.

#### Hepatic senescence-associated markers (*SA-β-Gal*, *p21* and *p16*)

**Fig. 8:**
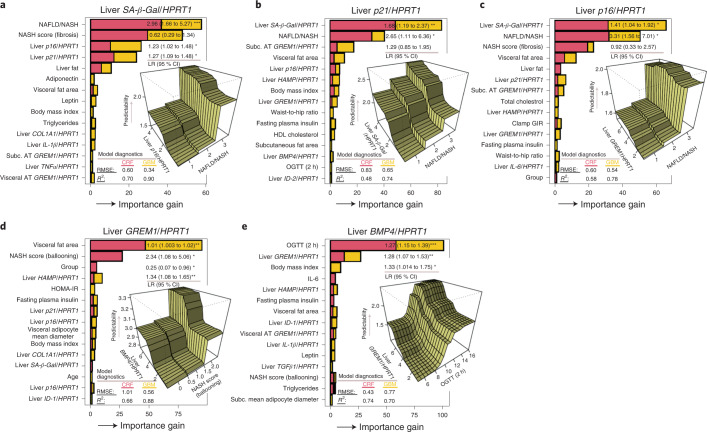
Strength of association of selected predictors for senescence markers and *GREM1* and *BMP4* mRNA levels in patients with NAFLD/NASH. **a**–**e**, Relative feature importance for senescence markers and *GREM1* and *BMP4* mRNA, using conditional random forest and gradient boosting model diagnostics, is presented in each panel. Predictors that display a pronounced increased in importance compared with other predictors are strong predictors for the outcome. Partial dependence plots are included to display interaction effects between features with strong predictability for the outcome.

In Fig. 8a, the strongest predictors were NAFLD/NASH and degree of fibrosis, followed by the other senescence-associated hepatic markers, *p21* and *p16*. Regression also revealed that these predictors were statistically significant. In Fig. 8b, *SA-β-Gal* and NAFLD/NASH were the strongest predictors for liver *p21*, followed by *GREM1*. A partial dependence plot revealed that *SA-β-Gal* increased prediction of *p21* in an incremental fashion (Fig. 8b), and liver *GREM1* demonstrated a strong interaction with NAFLD/NASH for prediction of liver *p16* (Fig. 8c).

#### Liver GREM1 and BMP4

In Fig. 8d, the most important predictor for GREM1 was visceral fat area, whereas degree of ballooning was important in the conditional random forest model, which demonstrated inferior model diagnostics (conditional random forest root mean squared error (r.m.s.e.), 0.56; gradient boosting model *R*^2^, 0.88). However, the linear regression model indicated that visceral fat area, degree of ballooning, group (that is, lean and obese) and liver *HAMP* were statistically significant.

In Fig. 8e, a prediction model for liver *BMP4* revealed that oral glucose tolerance test (OGTT; 2 h) (β, 1.27; 95% CI, 1.15 to 1.39), liver *GREM1* (β, 1.28; 95% CI, 1.07 to 1.53) and *COL1A1* (β, 1.33; 95% CI, 1.014 to 1.75) were the most important predictors. The partial dependence plot showed a strong interaction effect between liver *GREM*1 and OGTT for predicting liver *BMP4*.

Taken together, these independent analyses of available biomarkers identify senescence markers, visceral adipose tissue and *GREM1* as the most consistent predictors of the various components of NAFLD/NASH. However, markers of the total amount of adipose tissue were prominent for predicting the degree of steatosis and liver fat.

## Discussion

The present study in a large cohort of individuals with NAFLD/NASH clearly shows that the key markers of senescence, *SA-β-Gal*, *p16*, *p21* and *p53*, are increased in liver cells in NAFLD, further increased in NASH, and related to both amount of liver fat, as well as a pathologist’s grading of NAFLD and NASH. Although it is clear that obesity and increased amount of body fat are associated with liver steatosis, it was the amount of visceral fat, rather than the much larger subcutaneous fat depot, which was found by machine learning to be a major predictor of senescence, whereas free fatty acid levels did not appear to be as strongly predictive. This may indicate that visceral fat, the blood from which is drained via liver, may secrete factors that enhance the development of fibrosis and NASH. Although there are several possibilities, machine learning identified *GREM1* as a strong predictor of several components, including degree of fibrosis. GREM1 has been well characterized as a secreted antagonist that regulates BMP signaling via several mechanisms, including binding to BMP dimers and preventing engagement of BMP receptors, as well as sequestration of BMP4 precursor protein, thus preventing mature BMP4 secretion^47^. Our experimental studies also support a direct role of GREM1 in promoting cell senescence and antagonizing the anti-fibrotic and anti-inflammatory effects of BMP4. Indeed, we have previously shown that GREM1 is secreted by adipose and liver cells, is a circulating protein and has considerably higher expression in visceral tissue than in subcutaneous adipose tissue^48^. It inhibits the effect of insulin in liver and other target cells^48^, probably due to its BMP4-antagonistic effect, as BMP4 enhances whole-body insulin sensitivity, as well as insulin signaling and action in target cells^49,50^. Other visceral adipose tissue-associated factors that could be important are released pro-inflammatory factors; IL-6 has been shown to be elevated in portal blood compared with systemic levels^51^. We did not find visceral adipose tissue to have an increased percentage of senescent cells in NAFLD/NASH individuals, but as visceral fat was increased, overall senescent cell burden is increased, which could have promoted liver senescence via increased SASP factor secretion.

It is clear that both BMP4 and DOX, with antagonistic effects on cell senescence, affect the important YAP/TAZ signaling pathway. BMP4 increases the tumor suppressors LATS1, pTAZ and TAZ (an inhibitor of p53^33^) and has direct inhibitory effects on the CDK inhibitor p16. DOX, in turn, exerts completely opposite effects and reduces LATS1, pTAZ, TAZ, pYAP and YAP and increases p53. Interestingly, BMP4 has been shown to enhance reprogramming of human inducible pluripotent stem cells (iPSC) by lowering p16 through inhibitory effects by increased ID1–ID3^52^. On the other hand, GREM1 is primarily an antagonist of BMP4 but also seems to have direct enhancing effects on induction of senescence, with increased MDM2, which regulates p53 degradation, and increased p16. Thus, the cellular balance between BMP4 and GREM1 regulates ID1–ID3 and YAP/TAZ activation and effects on cellular senescence.

It is clear that obesity, insulin resistance and associated hyperinsulinemia are important drivers of NAFLD/NASH. We also found serum insulin levels to be correlated with degree of senescence, and insulin-like growth factor 1 (IGF-1) has been shown to increase senescence in human fibroblasts^53^, but there are no data about potential effects in human liver cells. Importantly, the degree of insulin resistance measured by the euglycemic clamp was very strongly associated with senescence, suggesting that factors associated with senescence (SASP factors) induce insulin resistance.

Our results support the degree of liver cell senescence as being a risk indicator for liver damage, including inflammation and fibrosis. However, a key question is if senescence drives this process or if it is mainly a marker of disease. One way of resolving this question is to examine what happens in patients with cancer treated with chemotherapy, and there is much information in the cancer literature. 5-fluorouracil (5-fu) is frequently used to treat different forms of cancer, and it has been shown that around 50% of the treated patients develop NAFLD with increased liver triglycerides and fibrosis^54^. Furthermore, studies in human primary liver cells have shown that 5-fu induces mitochondrial dysfunction as a mechanism for the increased lipid accumulation, as we also see here and has been reported in experimental mouse NAFLD models^6^. c-Jun N-terminal kinase (JNK), IL-8 and fibrosis markers are also activated by 5-fu^55^ similar to what is known in senescent cells. These clinical studies together with the current and other results support the contention that cell senescence is a primary driver of NAFLD/NASH.

Taken together, these studies show that human NAFLD/NASH is characterized by increased liver cell senescence that closely correlates with the degree of liver pathology and with the degree of insulin resistance. We found BMP4 to be an anti-senescent (but not senolytic) agent, whereas its inhibitor, GREM1, is pro-senescent, and the YAP/TAZ signaling pathway is a target of both pro-senescence and anti-senescence factors. The current results, together with clinical studies of the effect of chemotherapy in patients with cancer, suggest that liver senescence may be a primary factor driving development of NAFLD/NASH. Our results also point to BMP4 and anti-GREM1 therapy as being new avenues for preventing NAFLD/NASH and reducing senescence.

Although this is a very large translational study, it should be emphasized that an important weakness, like in all human studies of NAFLD/NASH, is the small number of healthy control participants available for comparing molecular data. A clinical indication is needed to perform liver biopsies in humans, but we can still evaluate the different stages of NAFLD/NASH development. In addition, currently available databases indicate that it is not just primary liver and stellate cells that become senescent, but also mesenchymal, endothelial and inflammatory cells. Analysis of single cells is needed to better understand this, but currently available data are too few to allow any firm conclusions.

## Methods

### Human participants

Paired samples of subcutaneous and visceral adipose tissue and liver were investigated in 58 individuals (27 females and 31 males, aged 31–90 years) who underwent elective surgical procedures (cholecystectomy, weight reduction or bariatric surgery, or exploratory laparotomy). Adipose tissue and liver biopsies were immediately snap-frozen in liquid nitrogen and stored at −80 °C until further analysis. The collection of tissues and human phenotyping was approved by the ethics committee of the University of Leipzig (Leipzig, Germany; approval no. 159-12-21052012), and all study participants gave written informed consent before taking part in the study. The participants received no financial compensation or gifts for participating. In brief, liver fat content (%) and abdominal visceral and subcutaneous fat area (cm^2^) were calculated using computed tomography or magnetic resonance imaging scans, whereas body fat content (%) was measured using dual-energy X-ray absorptiometry. Insulin sensitivity was assessed using the HOMA-IR index or by the euglycemic-hyperinsulinemic clamp method. All blood samples were collected between 8:00 and 10:00 after an overnight fast. Plasma insulin was measured with an enzyme immunometric assay by an Immulite automated analyzer (Diagnostic Products). Serum total cholesterol, high-density lipoprotein (HDL) cholesterol, low-density lipoprotein (LDL) cholesterol, free fatty acids, triglycerides, adiponectin and leptin (enzyme-linked immunosorbent assay (ELISA); Linco Research), IL-6 (ELISA; R&D Systems), plasma glucose and hemoglobin A1c (HbA1c; %) were determined. Adipo-IR was calculated as the product of free fatty acids (mmol l^−1^) and fasting insulin (pmol l^−1^)^56^. NAFLD and NASH were determined and diagnosed histologically by a certified pathologist following a strategy for grading and staging of the histological lesions detected in liver biopsies^57^.

### Liver tissue: gene mRNA expression analysis

RNA from human liver biopsy donors was extracted using RNeasy Lipid Tissue Mini Kits (Qiagen). The quantity and integrity of the RNA were monitored with a NanoVue Plus Spectrophotometer (GE Healthcare). A total of 1 µg of RNA was reverse-transcribed with standard reagents (Life Technologies). Complementary DNA was then processed for TaqMan probe-based quantitative real-time PCR (RT–qPCR) using a QuantStudio 6 Flex Real-Time PCR System (Life Technologies). The expression of target genes was calculated by the standard curve method and normalized to the expression of hypoxanthine-guanine phosphoribosyltransferase 1 (*HPRT1*) as a housekeeping gene. The primers and probes used are listed in Supplementary Table 5.

### RNA-seq analyses

#### Human adipose tissue

Total RNA from subcutaneous and visceral adipose tissue was extracted using RNeasy Lipid Tissue Mini Kits. RNA integrity was verified on an Agilent Bioanalyzer with the RNA nanochip. The RNA-seq libraries were prepared using an Illumina TruSeq RNA Sample Prep Kit following the manufacturer’s instructions. After library construction, all libraries were sequenced on an Illumina HiSeq platform as 101 bp paired-end reads to an average of 46.9 million reads per library. We used kallisto^58^ to estimate the count and number of transcripts per million (TPM) based on the Ensembl human reference genome (v102, GRCh38.p13). The sum value of the multiple protein-coding transcripts of a gene was used as the expression value of this gene. DESeq2 was used to identify the DEGs between groups^59^. KEGG functional enrichment analysis was performed using the R package ‘piano’, in which the log_2_[fold change] and *P* value of genes (visceral adipose tissue) from DESeq2 and gene annotation of KEGG pathways were used as input. We extracted significantly changed pathways based on the distinct-directional class in which the pathways are significantly affected by regulation in one distinct direction^60^. *P* < 0.05 was used to determine the significant dysregulated pathways. A complete list of the DEGs in visceral adipose tissue of NAFLD vs NASH is provided in Supplementary Table 6. The RNA-seq data of visceral adipose tissue used in this study can be downloaded from the Gene Expression Omnibus (GEO) database under accession number GSE200678.

#### IHH cells

Total RNA was isolated from the cells using E.Z.N.A. Total RNA Kits (Omega Bio-tek). RNA-seq libraries were prepared using Illumina RNA-Seq with Poly-A selections. Subsequently, the libraries were sequenced on a NovaSeq 6000 (NovaSeq Control Software 1.6.0/RNA v3.4.4) with a 2 × 51 setup using ‘NovaSeqXp’ workflow in ‘S1’ mode flow cell. The Bcl was converted to FastQ using bcl2fastq_v2.19.1.403 from the CASAVA software suite (Sanger/phred33/Illumina 1.8 + quality scale). The TPM and count values of genes were quantified in a similar way as described above. PCA was performed based on the genes with a mean TPM value >1 using the R package ‘factoextra’. DEseq2 was used to identify the DEGs between two groups of samples^59^. The *P* values were adjusted by the Benjamini–Hochberg method. Genes with a cutoff of adjusted *P* values (*P*_adj_) < 0.05 and fold change ≥ 1.2 were considered to be DEGs. KEGG functional enrichment analysis was performed using ‘piano’ as mentioned above. The RNA-seq data of cell lines used in this study can be downloaded from GEO under accession number GSE200679.

#### Liver tissue

Hepatic RNA-seq (raw FastQ files) of the NAFLD cohort included 10 normal samples, 50 patients with NAFLD and 155 patients with NASH^61^ retrieved from the European Nucleotide Archive database (https://www.ebi.ac.uk/ena) under accession number SRP217231. Gene abundance in both TPM and raw count were quantified using kallisto^58^ as described above. Differences among groups in gene TPM were detected by Mann–Whitney *U*-test, and *P* < 0.05 was considered statistically significant.

### Single-cell RNA-seq

Single-cell transcriptomic data were obtained from GEO under accession number GSE136103^30^. We extracted data from human samples and performed unsupervised clustering and differential gene expression analyses in the Seurat package (v4.0.0) following standard protocol^62^. We conducted differential gene expression analysis in Seurat using the Wilcoxon rank-sum test to assess significance, retaining only those genes with a log[fold change] of at least 0.2 and expression in at least 10% of cells in the cluster under comparison. All visualization was presented using Seurat functions.

### Cell culture

IHH cells and LX-2 (HSCs) cells (provided by J. Boren and S.L. Friedman, respectively) were cultured in William’s E medium (Life Technologies) and high-glucose Dulbecco’s Modified Eagle Medium (DMEM) (Life Technologies), respectively, supplemented with 10% fetal bovine serum (FBS) and 1% penicillin/streptomycin. DOX and etoposide were obtained from Sigma-Aldrich, and recombinant human proteins (TGF-β1, BMP4, Gremlin 1 and TNF-α) were from R&D Systems.

#### Cellular senescence induction in IHH cells

Cells were treated with different concentrations of DOX for 2 h in William’s E medium supplemented as described above. Cells were washed once and replenished with fresh media, followed by incubation for 72 h. As higher doses of DOX resulted in cell death, 2 μM was employed to delineate effects specific to senescence. We also collected the media after 72 h from both control cells (used as conditioned medium) and DOX-treated cells (used as senescent medium). The debris in conditioned medium and senescent medium was removed by centrifugation, and media were stored at −80 °C until use. To study the effects of BMP4 and GREM1, cells were incubated with different concentrations of recombinant BMP4 and GREM1 24 h prior to DOX treatment. After DOX treatment, cells were further incubated for 72 h in the presence of BMP4 or GREM1.

#### 3D spheroid culture

For generating cell spheroids, cells were seeded into 96-well round-bottom ultra-low attachment plates (Corning) at a density of 2,000 viable cells per well. IHH/LX-2 24:1 spheroids were grown in William’s E medium supplemented as described above. The plates were incubated for 48 h at 37 °C in a humidified atmosphere with 5% CO_2_. After 48 h, cells were treated with TGF-β1 or cocktail (oleic acid + TGF-β1 + TNF-α) with or without BMP4 or GREM1 for another 48 h.

#### LX-2 cells

LX-2 cells were seeded in DMEM containing 2% FBS. After 24 h, cells were treated with two different concentrations of TNF-α (0.2 nM and 0.4 nM), etoposide (1 µM and 2 µM) or DOX (25 nM or 50 nM), with or without BMP4 (25 ng ml^−1^), for 48 h. In addition, to study the effects of hepatocyte-secreted SASP factors, LX-2 cells were cultured for 48 h with conditioned medium and senescent medium (mixed in 50% (v/v) concentration) from IHH cells. Cells were collected for gene expression analysis, and supernatant was collected to determine the IL-1β levels using an ELISA (Thermo Fisher Scientific) according to the manufacturer’s instruction.

#### Preadipocytes, HUVECs and astrocytes

Abdominal subcutaneous adipose tissue for primary preadipocyte isolation was obtained during intra-abdominal surgery from healthy, lean participants undergoing surgery to donate a kidney (and given informed consent). The protocol was approved by the Mayo Clinic Foundation Institutional Review Board for Human Research. HUVECs (CC-2519) and astrocytes (CC-2565) were purchased from Lonza and grown according to the manufacturer’s protocol. Cells were radiated with 10 Gy to induce senescence or were sham-radiated. Twenty, ten, and fifteen days after radiation, 90% of cells were SA-β-Gal positive in preadipocyte, HUVEC and astrocyte cultures, respectively. Senescent and non-senescent cells were treated with different concentrations of BMP4, and cell viability was measured using a crystal violet assay. In brief, cells were fixed with 4% paraformaldehyde for 15 min at room temperature and then stained with 0.1% crystal violet for 30 min at room temperature. Room temperature was 22+/-2 degrees. Cells were washed, and staining intensity was measured at a wavelength of 540 nm using a multi-scan plate reader (Fisher).

### RNA interference

IHH cells were transfected with short interfering RNA (siRNA) directed against TAZ (SASI_Hs01_0012-4477) or MISSION siRNA Universal Negative Control (Sigma-Aldrich, SIC001). In brief, cells were transfected with 30 nmol l^−1^ siRNA using Lipofectamine RNAiMAX (Thermo Fisher Scientific) according to the manufacturer’s instructions.

### Quantitative real-time PCR

Total RNA was extracted from cells and tissues, followed by cDNA synthesis. Gene expression was then analyzed using a QuantStudio 6 Flex TaqMan System (Applied Biosystems). Relative expression was calculated using the ΔΔCt method with normalization to 18S ribosomal RNA or HPRT1. The primers and probes used are listed in Supplementary Table 5.

### Immunoblotting

Cells were washed with ice-cold PBS, and protein lysates were prepared using a lysis buffer. Protein concentration was determined using the Pierce BCA Protein Assay Kit (Thermo Fisher Scientific), and 20 μg of whole-cell extracts were loaded on NuPAGE Novex 4–12% Bis-Tris protein gels (Thermo Fisher Scientific). The Trans-Blot Turbo Transfer System (Bio-Rad) was used for protein transfer, and to ensure that the procedure had been fully completed, the membranes were colored using 0.5% Ponceau S (Merck Chemicals). The following antibodies were used: p21 (Santa Cruz Biotechnology, sc-6246), p53 (Cell Signaling Technology, 2527), p16 (Cell Signaling Technology, 18769), phospho-histone H2A.X (Cell Signaling Technology, 80312), β-galactosidase (Cell Signaling Technology, 27198), phospho-MDM2 (Cell Signaling Technology, 3521), MDM2 (Cell Signaling Technology, 86934), cleaved caspase-3 (Cell Signaling Technology, 9664), LATS1 (Cell Signaling Technology, 3477), LATS2 (Cell Signaling Technology, 5888), phospho-YAP (Cell Signaling Technology, 13008), phospho-TAZ (Cell Signaling Technology, 59971), YAP/TAZ (Cell Signaling Technology, 8418), pSMAD1/5/9 (Cell Signaling Technology, 13820) and glyceraldehyde-3-phosphate dehydrogenase (GAPDH; Santa Cruz Biotechnology, sc-47724), as well as the horseradish peroxidase (HRP)-conjugated secondary antibodies, anti-rabbit IgG (Cell Signaling Technology, 7074) and anti-mouse IgG (Cell Signaling Technology, 7076). Quantification was performed with normalization against loading controls.

### Immunocytochemistry

IHH cells grown on coverslips were treated with DOX and BMP4 or GREM1 as described above. Cells were fixed in 4% formaldehyde for 15 min and permeabilized in 0.1% Triton X-100 for 5 min. Cells were then blocked with 5% FBS for 1 h, followed by incubation with primary antibodies against Ki67 (Cell Signaling Technology, 9449), p21, p53, p16 and phospho-histone H2A.X for 1 h. Cells were then incubated with secondary antibody conjugated with Alexa Fluor 488 (Molecular Probes) for 1 h, nuclei were stained by 4,6-diamidino-2-phenylindole (DAPI) and the coverslip was mounted with fluorescence mounting medium (Invitrogen). Pictures were obtained using a Zeiss Axio Observer. Image analysis was performed using an in-house macro in ImageJ (v1.52h; National Institutes of Health, NIH), with nuclei being counted, the total area determined and a static threshold applied to all images for each of the fluorescent channels to determine positively stained area.

### SA-β-Gal staining

Cultured IHH cells were washed with PBS and fixed in SA-β-Gal stain fixing solution for 15 min at room temperature. Cells were then stained overnight with X-Gal solution using a commercial kit from Cell Signaling Technology (9860) according to the manufacturer’s directions and examined under a bright-field microscope (Zeiss Axio Vert).

### MitoTracker staining

For mitochondrial staining, cells were stained with 100 nM MitoTracker Red CMXRos (Thermo Fisher Scientific, M7512) for 30 min at 37 °C, followed by nuclear staining with DAPI (Sigma-Aldrich). Cells were washed and fixed in 4% (v/v) phosphate-buffered formaldehyde for 15 min at 37 °C. Images were taken using the Zeiss Axio Observer. Image analysis was performed using an in-house macro in ImageJ (v1.52h) as described above.

### Ultrastructural analysis

IHH cells were seeded in MatTek (MatTek Corporation), and ultrastructural analysis was performed using TEM. In brief, cells were fixed with Karnovsky solution (4% glutaraldehyde, 2% paraformaldehyde, 0.1 M cacodylate buffer (pH 7.4)) and subsequently post-fixed with buffered 1% osmium tetroxide and 1% potassium ferrocyanide in 0.1 M cacodylate buffer (pH 7.4), followed by staining with 1% uranyl acetate. Fixed cells were dehydrated through a graded series of ethanol and embedded in Hard Plus epoxy resin (Electron Microscopy Sciences). Ultrathin sections (70 nm) were cut and examined using a Talos 120 high-resolution transmission microscope (Thermo Fisher Scientific) at 12 kV.

### ORO staining

DOX-induced IHH cells (after 48 h) were incubated with different concentrations of oleic acid for 24 h. Cells were then fixed with 4% (v/v) phosphate-buffered formaldehyde (Histolab) and stained with 0.5% (wt/v) Oil Red O (Sigma-Aldrich) in 60% (v/v) isopropanol according to the manufacturer’s recommendation. To quantify intracellular lipid accumulation, ORO was eluted with 100% isopropanol and measured in a spectrophotometer at 492 nm. Cells were then stained with DAPI, and differences in cell number were corrected using DAPI fluorescence (measured at an excitation wavelength of 350 nm and an emission wavelength of 450 nm or 490 nm) to normalize the ORO signal in each well. Images were taken using a bright-field microscope (Zeiss Axio Vert).

### Spheroids and immunofluorescence

3D spheroids were fixed with 10% w/v paraformaldehyde (Sigma-Aldrich) for 2 h, then incubated with 20% w/v sucrose in PBS overnight, washed three times with PBS and embedded in OCT Cryomount (Histolab). Spheroids were sectioned into 8 µm thick slices using cryostat (Leica) and stored at −80 °C. For immunostaining, sections were incubated with 4% w/v bovine serum albumin (BSA; Sigma-Aldrich) in PBS for 1 h. Primary antibody COL1A1 (Cell Signaling Technology, 66948) and αSMA (Cell Signaling Technology, 19245) were diluted in 4% w/v BSA (PBS) and incubated for 1 h at room temperature, followed by washing and incubation with the fluorescent secondary antibodies Alexa Fluor 488 and 594 (Thermo Fisher Scientific, A-11001 and A-110012) for 1 h at room temperature. For ORO staining, sections were incubated with 2% (v/v) phosphate-buffered formaldehyde (Histolab) for 5 min. After 30 s of treatment with 20% (v/v) isopropanol, spheroids were stained with 0.5% (wt/v) ORO in 60% (v/v) isopropanol according to the manufacturer’s recommendation. Nuclei were stained by DAPI, and spheroids were mounted with fluorescence mounting medium (Dako). Pictures were obtained using Axioplan 2 (Zeiss) with AxioVision software (v4.8; Zeiss). Image analysis was performed using an in-house macro in ImageJ (v1.52h) in which nuclei were counted, the total spheroid area was determined and a static threshold was applied to all images for each of the fluorescent channels to determine positively stained area.

### Statistical analysis

All values are expressed as mean ± s.e.m. of at least three independent experiments as indicated in the figure legends. Depending on the experimental design, statistical significance was determined by unpaired *t*-test, Mann–Whitney tests when appropriate or one-way analysis of variance (ANOVA) with post hoc tests as appropriate. To assess correlation between variables, Pearson or Spearman correlations were used as appropriate. All statistical tests were performed using GraphPad Prism 9.0 and R Studio (v4.0.3) for machine learning analyses. Significance is indicated in the figures as *P* < 0.05, *P* < 0.01 and *P* < 0.001.

### Predictive models

Conditional random forest, gradient boosting and ordinary random forest were applied to estimate relative feature importance and interactions. Prediction models were also used as a feature selection process prior to multiple regression for estimation of effect size for features. Results from conditional random forest were compared with those of gradient boosting, which (in contrast to conditional random forest) grows each decision tree on the residuals of the previous tree, and prediction is performed differentially.

### Model building

Hyperparameter optimization was performed by automated grid search. Parameters for conditional random forest model included number of trees, predictors in each split, minimum sum of weights in a node to be considered for splitting and the proportion of observations needed to establish a terminal node. Parameters for gradient boosting models included interaction depth, number of trees, shrinkage factor and number of observations in a terminal node. Each model was validated with repeated cross-validation using five to ten iterations. Prediction models for each outcome contain a unique subset of predictors, as features with strong correlations were excluded prior to model building.

### Partial dependence plots

Interaction effects between strong predictors were assessed with two-way partial dependence plots, based on ordinary random forest models. These figures are presented beside each prediction model and demonstrate the interaction effects of varying values for the most important predictors.

### Logistic and linear regression

Features with greatest relative importance in prediction models were subsequently included in linear and logistic regression models for assessment of effect size and significance level. The regression estimates and 95% CIs are presented next to each prediction model in the figures.

### Elastic net regression

For variables related to insulin sensitivity, we used elastic net regression as feature selection for linear regression models, and ten folds for cross-validation. Features that demonstrated importance were subsequently included in a linear regression model. The reason for using elastic net for feature selection was due to poor model diagnostics for insulin sensitivity-related variables from the predictive machine learning models.

### Imputation

We used a random forest algorithm to impute missing data for study participants^63^. We analyzed distributions and means before and after imputation without observing any differences. *P* values < 0.05 were considered significant.

Calculations were performed in R (v4.0.2) using the following machine learning libraries: Corrplot, GBM, missForest, Random Forest, Caret, ggRandomForests, Party, Plotmo, GridExtra, cForest, MLR, coefplot, glmnet and hydroGOF.

### Reporting summary

Further information on research design is available in the Nature Research Reporting Summary linked to this article.

## Supplementary information

Supplementary Information Supplementary Figs. 1–3

Reporting Summary

Supplementary Tables Supplementary Tables 1–6

Supplementary Data Statistical source data for Supplementary Figs. 1 and 2

## Data Availability

All information is included in the manuscript (and its Supplementary Information). All figures and Extended Data figures have associated raw data that is provided as an Excel worksheet. Transcriptomic data for visceral adipose tissue and cell lines were deposited in GEO under accession numbers GSE200678 and GSE200679, respectively. The publicly available sequencing data used in the study were retrieved from European Nucleotide Archive database (SRP217231) and from GEO (GSE136103). Source data are provided with this paper.

## Source data

Source Data Fig. 1 Statistical source data.
Source Data Fig. 2 Statistical source data.
Source Data Fig. 3 Statistical source data.
Source Data Fig. 4 Statistical source data.
Source Data Fig. 4 Unprocessed western blots.
Source Data Fig. 5 Statistical source data.
Source Data Fig. 5 Unprocessed western blots.
Source Data Fig. 6 Statistical source data.
Source Data Extended Data Fig. 1 Statistical source data.
Source Data Extended Data Fig. 2 Statistical source data.
Source Data Extended Data Fig. 3 Statistical source data.
Source Data Extended Data Fig. 3 Unprocessed western blots.
Source Data Extended Data Fig. 4 Statistical source data.
Source Data Extended Data Fig. 5 Statistical source data.
Source Data Extended Data Fig. 6 Statistical source data.
Source Data Extended Data Fig. 7 Statistical source data.

## Code availability

All computer codes used to generate results reported in the manuscript are available upon request.
