# Supplementary material for: BMP4 and Gremlin 1 regulate hepatic cell senescence during clinical progression of NAFLD/NASH
Source: Nat Metab. 2022 Aug 22;4(8):1007–21. doi: 10.1038/s42255-022-00620-x (PMC9398907; doi:10.1038/s42255-022-00620-x)
Supplement: Supplementary file 1 — Supplementary Figs. 1–3 [file 42255_2022_620_MOESM1_ESM.pdf]

nature portfolio

https://doi.org/10.1038/s42255-022-00620-x

Supplementary information

BMP4 and Gremlin 1 regulate hepatic cell senescence during clinical progression of NAFLD/NASH

In the format provided by the authors and unedited

Supplementary Figures

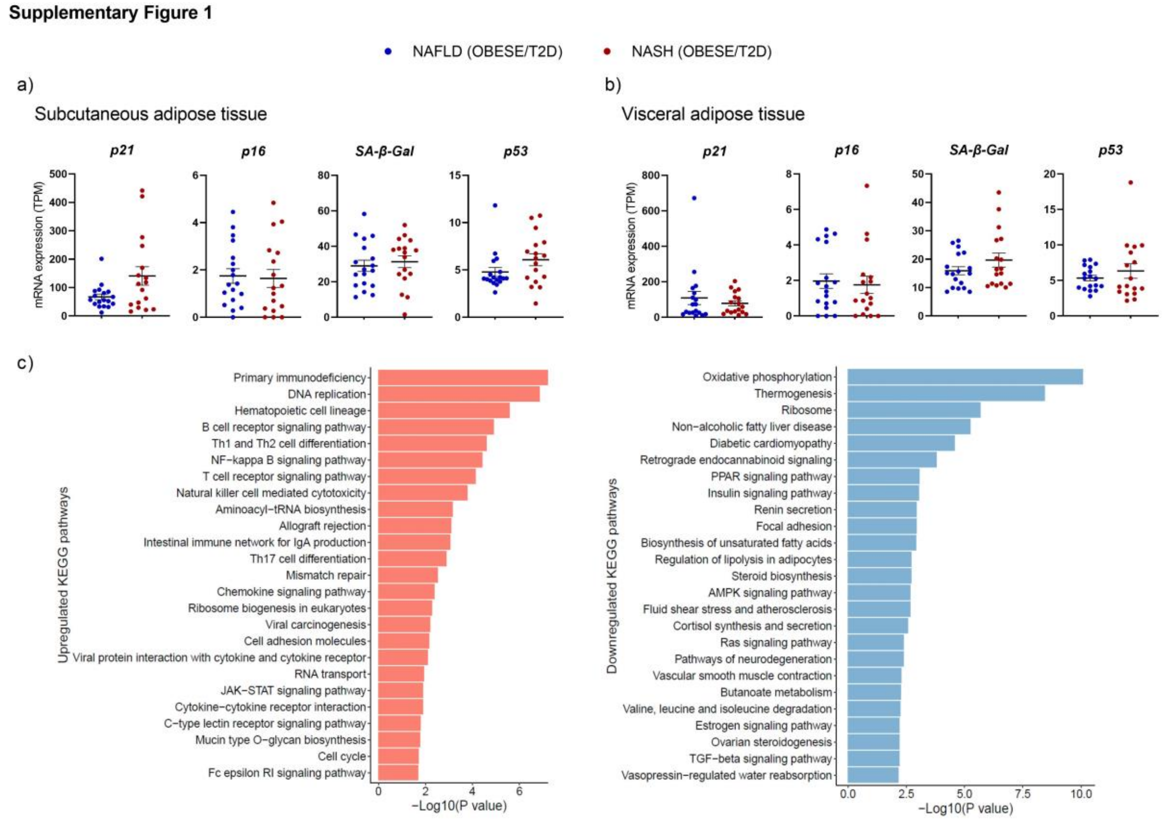

Supplementary Figure 1: (a, b) Expression of senescence markers (*p21*, *p16*, *SA-β-Gal* and *p53*) in subcutaneous (a) and visceral adipose (b) tissue of NAFLD (n=18) and NASH (n = 17) individuals. RNA-seq data are shown as transcripts per million (TPM). Values are mean ± SEM. Statistics were calculated using 2-tailed, unpaired t-test or Mann-Whitney test. (c) KEGG pathway enrichment analysis of differentially expressed upregulated and downregulated genes from visceral adipose tissue of NAFLD and NASH individuals. Two-tailed p-values in distinct-directional class (up-regulation or down-regulation) were calculated from a theoretical null-distribution by using R package "piano". $p < 0.05$ was considered to indicate a statistically significant difference.

Supplementary Figure 2

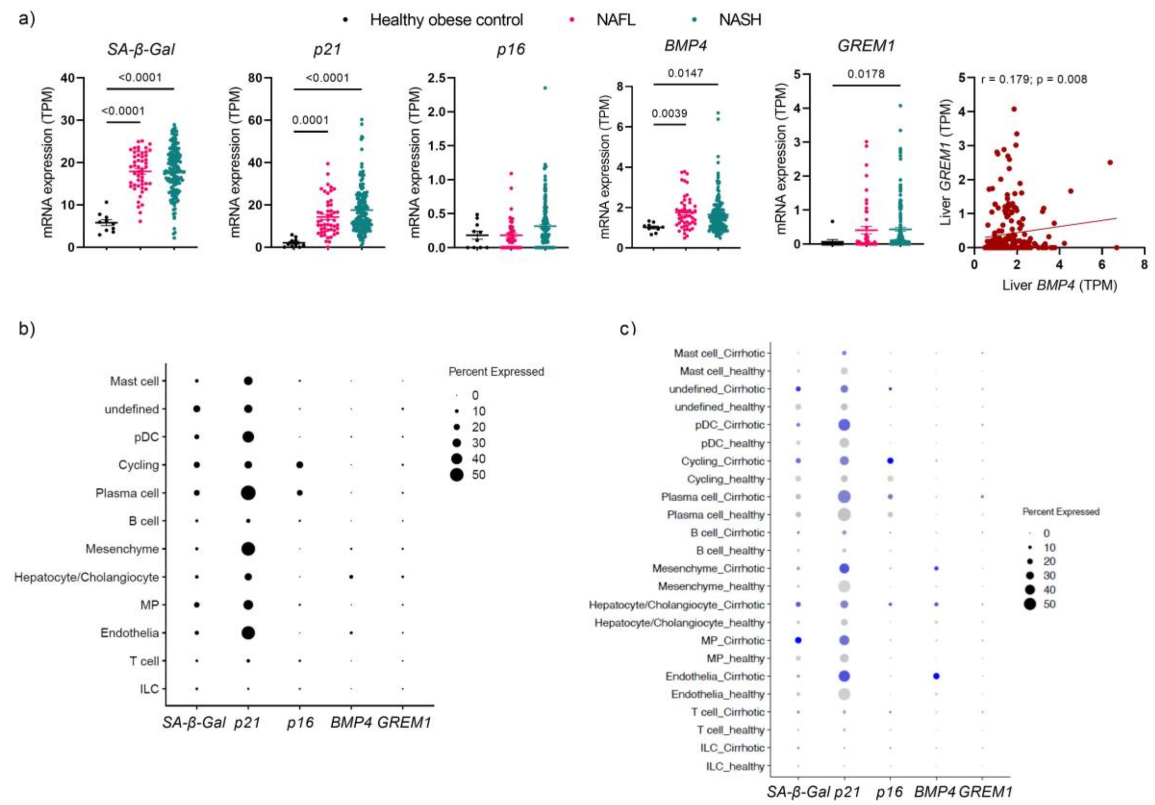

Supplementary Figure 2: (a) Hepatic expression levels of senescence markers (*p21*, *p16*, *SA-β-Gal*), *BMP4* and *GREM1* in healthy obese (n = 10), NAFL (n = 50) and NASH (n = 155) individuals (publically available dataset[61]). RNA-seq data are shown as transcripts per million (TPM). Values are mean ± SEM. Statistical significance was determined by one-way ANOVA with *post-hoc* Tukey's test or Kruskal-Wallis with *post-hoc* Dunn's test. (b, c) Gene expression analysis of publically available scRNA-seq dataset[30]: Expression of indicated genes across the 11 cell types in healthy liver samples (b), as well as in cirrhotic liver samples (along with expression in healthy liver samples) (c). The size of the dot corresponds to the percentage of cells expressing the gene in each cell type, the color represents the average expression level. MP, mononuclear phagocyte; pDC, plasmacytoid dendritic cell; ILC, innate lymphoid cell.

Supplementary figure 3

Graphical abstract

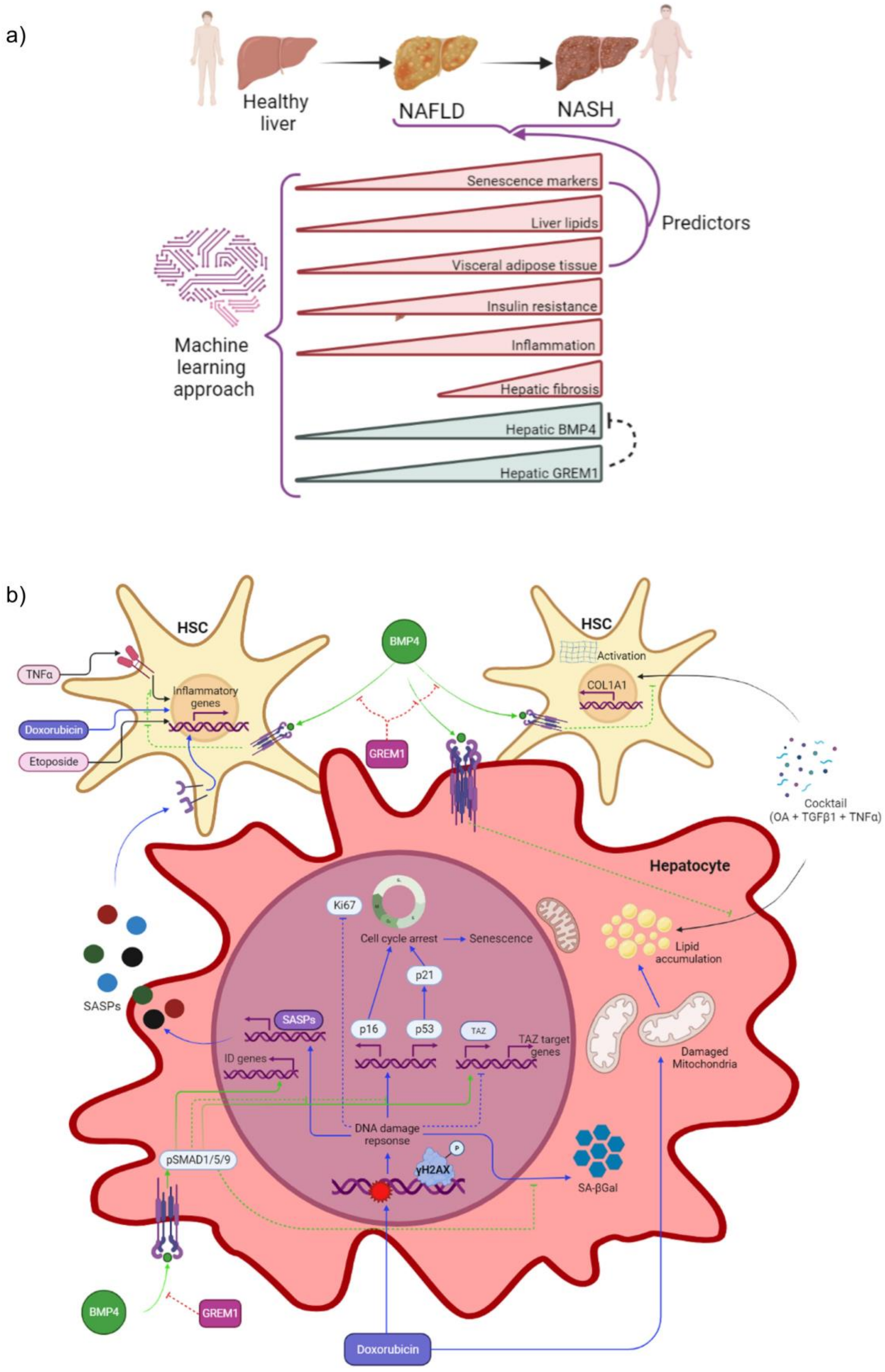

Supplementary Figure 3: Graphical abstract shows increased hepatic senescence in NAFLD/NASH and identified mechanisms of BMP4 (and GREM1) against doxorubicin-induced senescence. (a) Senescence markers are increased in the liver of NAFLD/NASH patients with increasing severity of disease. Their increase is closely associated with increase in liver lipids, visceral adipose tissue, insulin resistance, inflammation as well as hepatic fibrosis. Additionally, increase in hepatic BMP4 and its antagonist GREM1 is also associated with increase in hepatic senescence markers. Interestingly, Machine-Learning approach identifies senescence markers, amount of visceral adipose tissue and GREM1 expression as important predictors of disease development. (b) In vitro model system showing human hepatocytes undergo senescence in response to doxorubicin, a DNA-damaging agent. Mechanistically, doxorubicin leads to increase in γH2AX as a result of DNA-damage, which in turn activates the p53-p21 and the p16 pathways, driving cell cycle arrest (reduces Ki67) and senescence (increases SA-βGal activity). Doxorubicin also triggers TAZ downregulation, trans-activating the p53-p21 pathway. Additionally, doxorubicin induces mitochondrial damage leading to reduced oxidation and lipid accumulation as well as enhanced SASPs secretion in hepatocytes resulting in increased expression of inflammatory genes in hepatic stellate cells (HSCs). Intriguingly, BMP4 is antagonistic to the doxorubicin-mediated increase in p53 & p16, reduces expression of pro-inflammatory cytokines and enhances TAZ and its target genes in hepatocytes. BMP4 also prevents lipid accumulation in hepatocytes exposed to a cocktail of lipogenic & inflammatory triggers. HSCs, playing a key role in liver fibrosis, increases fibrotic markers (COL1A1 & αSMA) when exposed to TGFβ1, or increases pro-inflammatory markers when exposed to inflammatory triggers (e.g.; TNFα) or senescence-inducing agents (doxorubicin or etoposide). BMP4 reduces both fibrotic as well as inflammatory markers in HSCs exposed to different insults. GREM1, on the other hand, works via inhibiting BMP4-SMAD signaling that results in downregulation of its downstream *Id* genes. GREM1 promotes senescence in hepatocytes and prevents the anti-inflammatory effects of BMP4 in HSCs.

Solid lines with arrows represent activation and dotted lines represent inhibition. Blue lines indicate the effects of doxorubicin, green lines indicate the effects of BMP4 and red lines indicate the effects of GREM1. (Figure has been created with BioRender.com)
