# Supplementary material for: BMP4 and Gremlin 1 regulate hepatic cell senescence during clinical progression of NAFLD/NASH
Source: Nat Metab. 2022 Aug 22;4(8):1007–21. doi: 10.1038/s42255-022-00620-x (PMC9398907; doi:10.1038/s42255-022-00620-x)
Supplement: Supplementary file 2 — Reporting Summary [file 42255_2022_620_MOESM2_ESM.pdf]

nature research

Corresponding author(s): ULF SMITH

Last updated by author(s): Jul 1, 2022

Reporting Summary

Nature Research wishes to improve the reproducibility of the work that we publish. This form provides structure for consistency and transparency in reporting. For further information on Nature Research policies, see our Editorial Policies and the Editorial Policy Checklist.

Statistics

For all statistical analyses, confirm that the following items are present in the figure legend, table legend, main text, or Methods section.

n/a	Confirmed	
☐	☒	The exact sample size (*n*) for each experimental group/condition, given as a discrete number and unit of measurement
☐	☒	A statement on whether measurements were taken from distinct samples or whether the same sample was measured repeatedly
☐	☒	The statistical test(s) used AND whether they are one- or two-sided<br>*Only common tests should be described solely by name; describe more complex techniques in the Methods section.*
☒	☐	A description of all covariates tested
☐	☒	A description of any assumptions or corrections, such as tests of normality and adjustment for multiple comparisons
☐	☒	A full description of the statistical parameters including central tendency (e.g. means) or other basic estimates (e.g. regression coefficient) AND variation (e.g. standard deviation) or associated estimates of uncertainty (e.g. confidence intervals)
☐	☒	For null hypothesis testing, the test statistic (e.g. *F*, *t*, *r*) with confidence intervals, effect sizes, degrees of freedom and *P* value noted<br>*Give P values as exact values whenever suitable.*
☒	☐	For Bayesian analysis, information on the choice of priors and Markov chain Monte Carlo settings
☒	☐	For hierarchical and complex designs, identification of the appropriate level for tests and full reporting of outcomes
☐	☒	Estimates of effect sizes (e.g. Cohen's *d*, Pearson's *r*), indicating how they were calculated

Our web collection on statistics for biologists contains articles on many of the points above.

Software and code

Policy information about availability of computer code

Data collection	Images for cells (2D) were acquired using a Zeiss Axio Observer. Spheroids (3D) pictures were obtained using Axioplan 2 (Zeiss) with AxioVision 4.8 Software (Zeiss). TEM images were acquired using high resolution transmission microscope Talos120 (ThermoFisher). RT-PCR data were collected using a Quant Studio 6 Flex TaqMan system (Applied Biosystems).). All publicly available data referenced in our manuscript have been collected from European Nucleotide Archive database (https://www.ebi.ac.uk/ena/) under accession number SRP217231 and Gene Expression Omnibus database with accession number GSE136103.
Data analysis	Statistical analysis was performed using GraphPad Prism 9.0 (GraphPad Software) and R Studio v 4.0.3 for machine learning analysis. Western blotting Image quantification was performed with Image Lab V6 (Biorad). Fluorescent image analysis was performed using an in-house macro in ImageJ (v.1.52h, NIH). DESeq2 was used to identify the DEGs between groups and R package 'piano' was used for KEGG functional enrichment analysis. All computer code used to generate (Machine Learning) results reported in the manuscript are available upon request.

For manuscripts utilizing custom algorithms or software that are central to the research but not yet described in published literature, software must be made available to editors and reviewers. We strongly encourage code deposition in a community repository (e.g. GitHub). See the Nature Research guidelines for submitting code & software for further information.

Data

Policy information about availability of data

All manuscripts must include a data availability statement. This statement should provide the following information, where applicable:

- Accession codes, unique identifiers, or web links for publicly available datasets
- A list of figures that have associated raw data
- A description of any restrictions on data availability

All the information is included in the manuscript (and its supplementary information files). All Main and Extended Figures have associated raw data that is provided

as an Excel worksheet. Transcriptomic data for visceral AT and cell lines was deposited in GEO with accession number GSE200678 and GSE200679, respectively. The publically available sequencing data used in the study was retrieved from European Nucleotide Archive database (SRP217231) and from Gene Expression Omnibus database (GSE136103). All computer codes used in this study are available from the corresponding authors upon reasonable request.

Field-specific reporting

Please select the one below that is the best fit for your research. If you are not sure, read the appropriate sections before making your selection.

[x] Life sciences [ ] Behavioural & social sciences [ ] Ecological, evolutionary & environmental sciences

For a reference copy of the document with all sections, see nature.com/documents/nr-reporting-summary-flat.pdf

Life sciences study design

All studies must disclose on these points even when the disclosure is negative.

Sample size	No statistical method was used to determine sample sizes but sample size used was based on the results from our previous studies (PMID: 30067159; PMID: 34426590; PMID: 31227697; PMID: 31882566). For invitro experiments, at least three biological replicates were used for each experiment to ensure the reproducibility and to perform statistical analysis.
Data exclusions	No data was excluded, unless there is failure in measurement.
Replication	Only unique human samples were used, so replication was not possible. For invitro experiment, data was obtained from at least three biological replicates and for each series of experiments, all replication attempts were successful.
Randomization	Patients grouping was based on clinical characteristics, and as such no randomization was performed. For invitro experiments, experimental groups were randomized only to the extent that the sample groups were distributed across cell culture plates in order to avoid positional artefacts such as edge effects.
Blinding	The experiments were performed and analyzed in both blinded and non-blinded fashions, where applicable. ORO and IHC staining analysis (staining and scoring) was done in a blinded fashion. For other experiments, data points were obtained using standardized equipment and assay kits (RT-qPCR, immunoassay etc.) and therefore, we did not perform blind test.

Reporting for specific materials, systems and methods

We require information from authors about some types of materials, experimental systems and methods used in many studies. Here, indicate whether each material, system or method listed is relevant to your study. If you are not sure if a list item applies to your research, read the appropriate section before selecting a response.

Materials & experimental systems

n/a	Involved in the study	
[ ]	[x]	Antibodies
[ ]	[x]	Eukaryotic cell lines
[x]	[ ]	Palaeontology and archaeology
[x]	[ ]	Animals and other organisms
[ ]	[x]	Human research participants
[x]	[ ]	Clinical data
[x]	[ ]	Dual use research of concern

Methods

n/a	Involved in the study	
[x]	[ ]	ChIP-seq
[x]	[ ]	Flow cytometry
[x]	[ ]	MRI-based neuroimaging

Antibodies

Antibodies used

The following antibodies were used:
For WB:
p53 (7F5) (Cell Signaling Technology; 2527; 1:1000)
p16 INK4A (E6N8P) (Cell Signaling Technology; 18769; 1:1000)
p21 Waf1/Cip1/CDKN1A Antibody (F-5) (Santa Cruz Biotechnology; sc-6246; 1:1000)
Phospho-Histone H2A.X (Ser139) (D7T2V) (Cell Signaling Technology; 80312; 1:1000)
β-Galactosidase (E2U2I) (Cell Sinaling Technology; 27198; 1:1000)
Phospho-MDM2 (Ser166) (Cell Signaling Technology; 3521; 1:1000)
MDM2 (D1V2Z) (Cell Signaling Technology; 86934; 1:1000)
Cleaved Caspase-3 (Asp175) (5A1E) (Cell Signaling Technology; 9664; 1:1000)
LATS1 (C66B5) (Cell Signaling Technology; 3477; 1:1000)
LATS2 (D83D6) (Cell Signaling Technology; 5888; 1:1000)
Phospho-YAP (Ser127) (D9W2I) (Cell Signaling Technology; 13008; 1:1000)
Phospho-TAZ (Ser89) (E1X9C) (Cell Signaling Technology; 59971; 1:1000)

YAP/TAZ (D24E4) (Cell Signaling Technology; 8418; 1:1000)
pSMAD1/5/9 (Cell Signaling Technology; 13820; 1:1000)
GAPDH Antibody (0411) (Santa Cruz Biotechnology; sc-47724; 1:1000)
Anti-rabbit IgG, HRP-linked Antibody (Cell Signaling Technology; 7074; 1:2000)
Anti-mouse IgG, HRP-linked Antibody (Cell Signaling Technology; 7076; 1:2000).

For IF:
COL1A1 (Cell Signaling; 66948; 1:200)
αSMA (Cell Signaling; 19245; 1:100)
Alexa Fluor 594 (Thermo Fischer Scientific; A-11012; 2 μg/mL)
Alexa Fluor 488 (Thermo Fischer Scientific; A-11001; 1 μg/mL)

Validation

All antibodies are from commercial sources. They are all commercial antibodies with validations available either as proofs or publication references on the manufacturers website.

Cell Signaling: https://www.cellsignal.com/about-us/cst-antibody-validation-principles
"To ensure our antibodies will work in your experiment, we adhere to the Hallmarks of Antibody Validation™, six complementary strategies that can be used to determine the functionality, specificity, and sensitivity of an antibody in any given assay. CST adapted the work by Uhlen, et. al., ("A Proposal for Validation of Antibodies." Nature Methods (2016)) to build the Hallmarks of Antibody Validation, based on our decades of experience as an antibody manufacturer and our dedication to reproducible science."

Eukaryotic cell lines

Policy information about cell lines

Cell line source(s)

IHH cells (PMID: 9298258) - a kind gift from Prof. Jan Boren
LX2 cells- a kind gift from Prof. Scott. L. Friedman
Preadipocytes (described in methods)
HUVECs (Lonza, CC-2519)
Normal Human Astrocytes (Lonza; CC-2565)

Authentication

Authenticated by the providers.
IHH cells (authenticated based on liver cell markers and cell morphology; PMID: 9298258)
LX2 cells (identity has been validated by PCR, IHC and functional assays; PMID: 15591520)
Preadipocytes (identity has been validated by PCR and routine observation of cell morphology; PMID: 16936206)
HUVECs (identity has been validated by PCR and routine observation of cell morphology)
Normal Human Astrocytes (identity has been validated by PCR and routine observation of cell morphology)

Mycoplasma contamination

Cell lines used in the article were tested for mycoplasma contamination. All the cells were mycoplasma-negative.

Commonly misidentified lines
(See ICLAC register)

No commonly misidentified cell lines were used in the study,

Human research participants

Policy information about studies involving human research participants

Population characteristics

Paired samples of subcutaneous and visceral AT, and liver were investigated in 58 individuals (27 females & 31 males, age ranged from 31 to 90 years). The detailed population characteristics of the human research participants were described in Supplementary Table 1. All study participants gave written informed consent before taking part in the study. The participants received no financial compensation or gifts for participating.

Recruitment

Individuals who underwent elective surgical procedures (cholecystectomy, weight reduction/bariatric surgery or exploratory laparotomy) were recruited.

Ethics oversight

Ethics committee of the University of Leipzig (approval number 159–12–21052012; Leipzig, Germany)

Note that full information on the approval of the study protocol must also be provided in the manuscript.
