# Supplementary material for: BMP4 and Gremlin 1 regulate hepatic cell senescence during clinical progression of NAFLD/NASH
Source: Nat Metab. 2022 Aug 22;4(8):1007–21. doi: 10.1038/s42255-022-00620-x (PMC9398907; doi:10.1038/s42255-022-00620-x)

Figure 4 : Doxorubicin induced senescence in IHH cells.

Corresponding uncropped blots for Fig. 4b

Sample No.	Details
1	Control
2	DOX 0.5 µM
3	DOX 1 µM
4	DOX 2 µM
5	DOX 4 µM

PageRuler Prestained Protein ladder (ThermoFisher Scientific; 26619) was used as size marker.

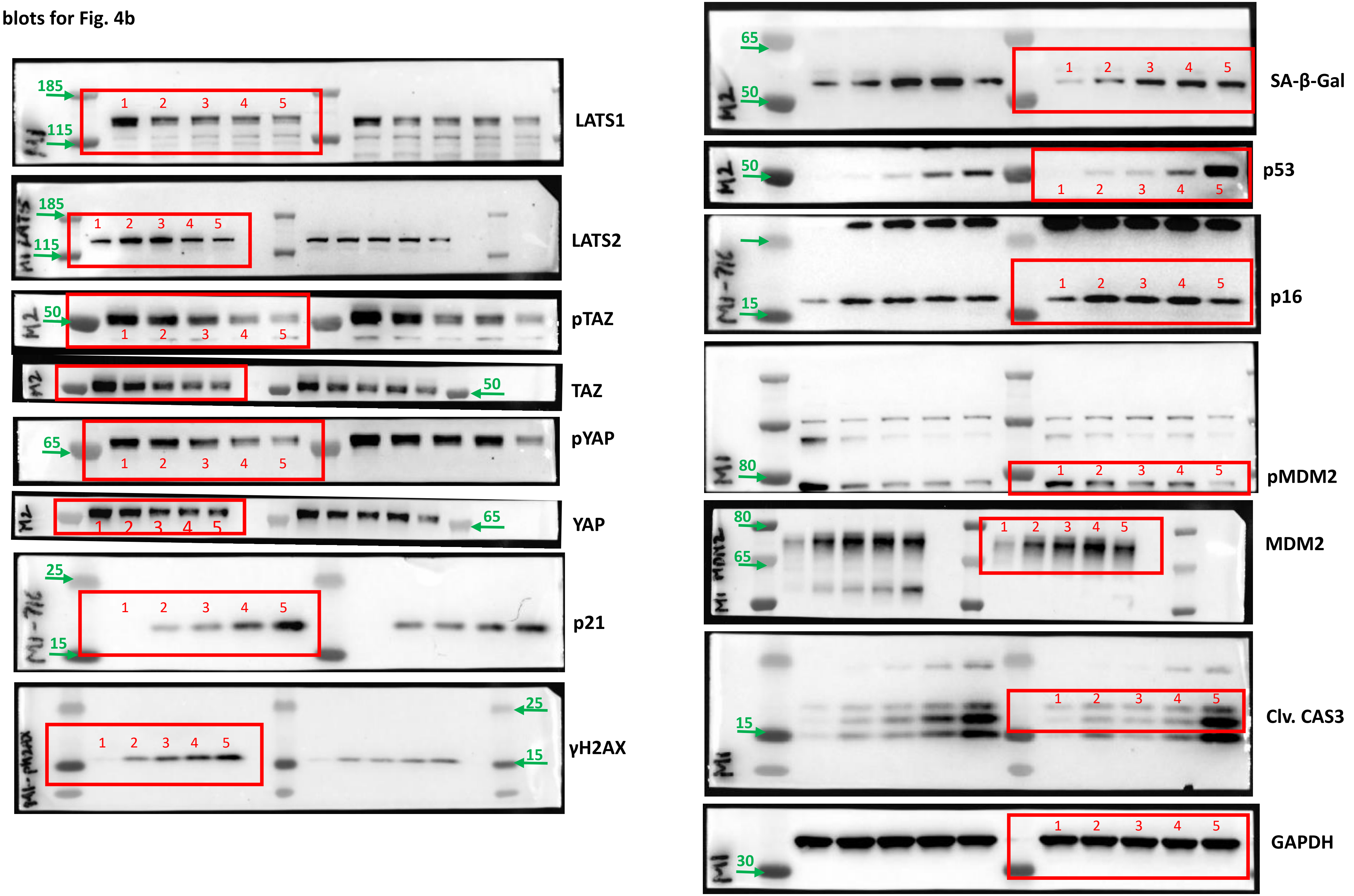

Supplement: Source Data Fig. 4 — Unprocessed western blots. [file 42255_2022_620_MOESM9_ESM.pdf]
