# Supplementary material for: BMP4 and Gremlin 1 regulate hepatic cell senescence during clinical progression of NAFLD/NASH
Source: Nat Metab. 2022 Aug 22;4(8):1007–21. doi: 10.1038/s42255-022-00620-x (PMC9398907; doi:10.1038/s42255-022-00620-x)
Supplement: Source Data Fig. 5 — Unprocessed western blots. [file 42255_2022_620_MOESM11_ESM.pdf]

Figure 5 : BMP4 prevented the increase in DOX-induced senescence markers, whereas GREM1 enhanced the effects of DOX

Corresponding uncropped blots for Fig. 5a

Sample No.	Details
1	Control
2	BMP4 (20ng/ml)
3	BMP4 (50ng/ml)
4	DOX 2 μM
5	DOX 2 μM + BMP4 (20ng/ml)
6	DOX 2 μM + BMP4 (50ng/ml)

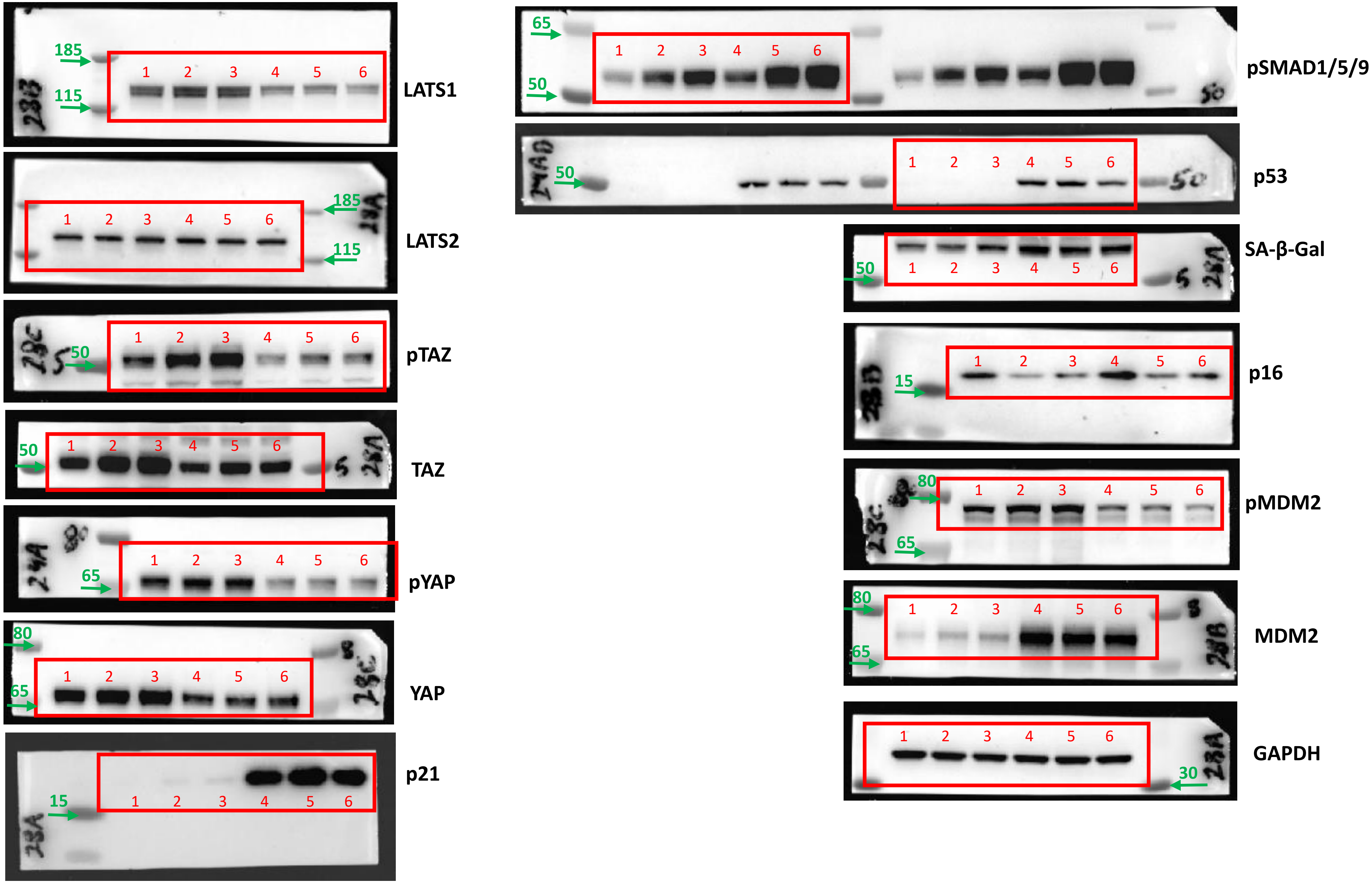

PageRuler Prestained Protein ladder (ThermoFisher Scientific; 26619) was used as size marker.

Figure 5 : BMP4 prevented the increase in DOX-induced senescence markers, whereas GREM1 enhanced the effects of DOX

Corresponding uncropped blots for Fig. 5c

Sample No.	Details
1	Control
2	GREM1 (200 ng/ml)
3	DOX 1 µM
4	DOX 1 µM + GREM1 (200 ng/ml)
5	DOX 2 µM
6	DOX 2 µM + GREM1 (200 ng/ml)

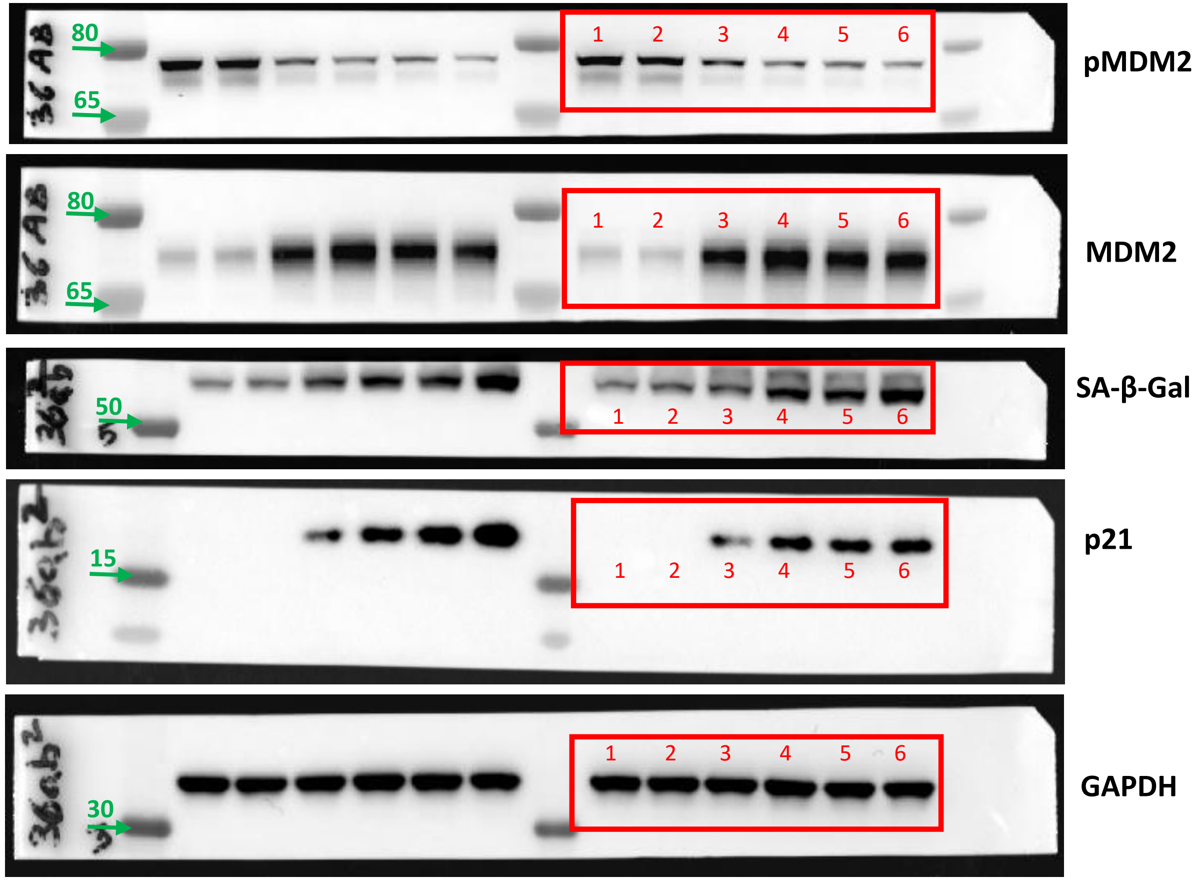

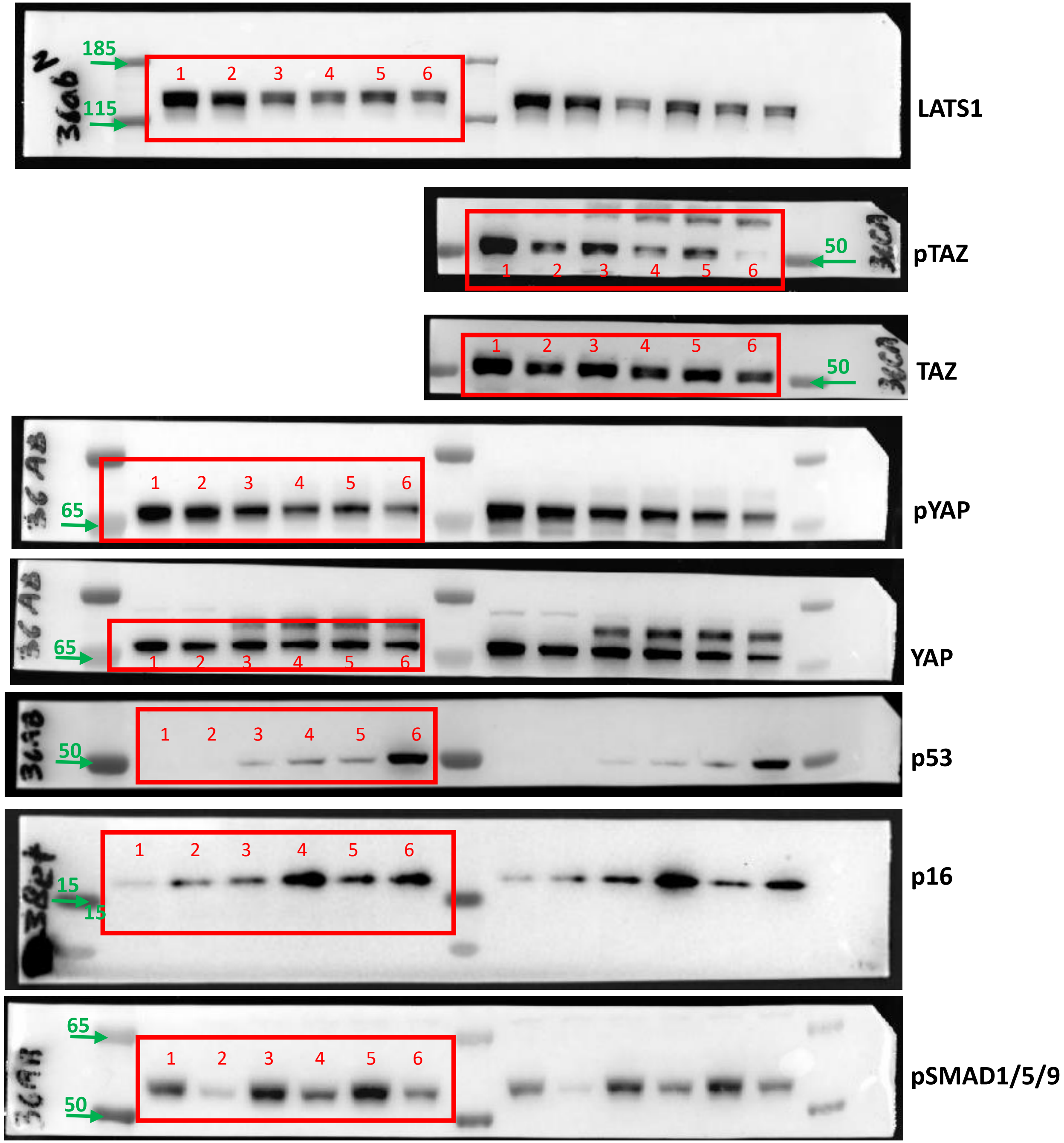

PageRuler Prestained Protein ladder (ThermoFisher Scientific; 26619) was used as size marker.
