# Supplementary material for: BMP4 and Gremlin 1 regulate hepatic cell senescence during clinical progression of NAFLD/NASH
Source: Nat Metab. 2022 Aug 22;4(8):1007–21. doi: 10.1038/s42255-022-00620-x (PMC9398907; doi:10.1038/s42255-022-00620-x)
Supplement: Source Data Extended Data Fig. 3 — Unprocessed western blots. [file 42255_2022_620_MOESM16_ESM.pdf]

Extended data 3:

Corresponding uncropped blots for Extended data 3b

Sample No.	Details
1	Cells transfected with Scrambled siRNA
2	Cells transfected with TAZ siRNA

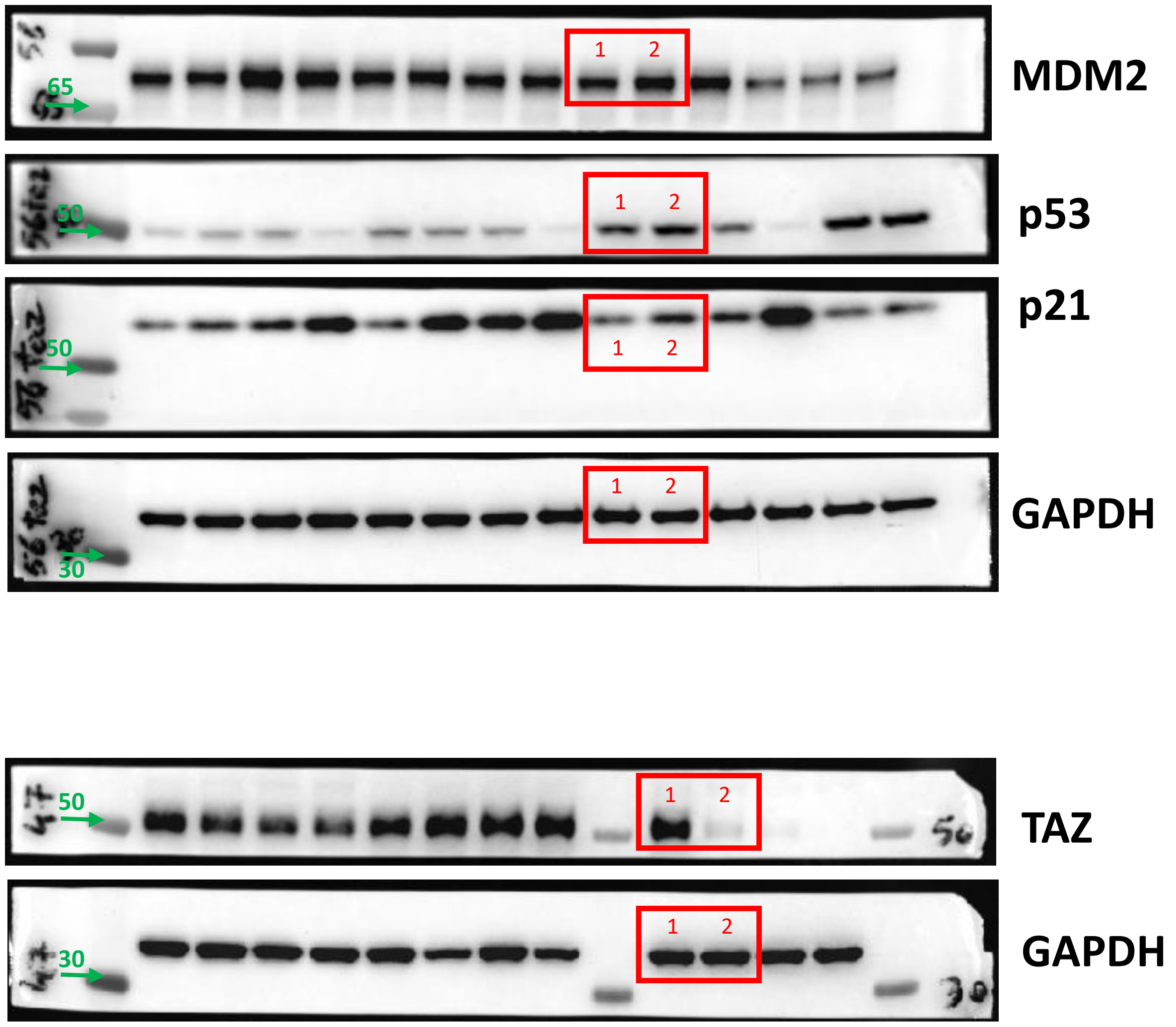

PageRuler Prestained Protein ladder (ThermoFisher Scientific; 26619) was used as size marker.
